# Early parasitological response following artemisinin-containing regimens: a critical review of the literature

**DOI:** 10.1186/1475-2875-12-125

**Published:** 2013-04-19

**Authors:** Debashish Das, Ric N Price, Delia Bethell, Philippe J Guerin, Kasia Stepniewska

**Affiliations:** 1 WorldWide Antimalarial Resistance Network (WWARN), Oxford, UK; 2 Centre for Tropical Medicine, Nuffield Department of Clinical Medicine, University of Oxford, Oxford, OX3 7LE, UK; 3 Global Health Division, Menzies School of Health Research and Charles Darwin University, Darwin, NT, Australia

**Keywords:** Artemisinins, Malaria, Plasmodium falciparum, Resistance

## Abstract

**Background:**

Parasitaemia on Day 3 has been proposed as a useful alert of potential artemisinin resistance, however, the normal variation of parasite clearance observed in artemisinin-based combination therapy clinical trials is poorly documented.

**Methods:**

The trends in early parasitological response following treatment with an artemisinin anti-malarial regimen were reviewed. A PubMed literature search identified all studies using an artemisinin regimen for uncomplicated falciparum malaria published between January 2000 and December 2011. Data from clinical studies were extracted for analysis using a standardized questionnaire.

**Results:**

In total 65,078 patients were enrolled into 213 clinical trials with 413 treatment arms containing either an artemisinin derivative alone (n=26) or in combination with a partner drug (n=387). The proportion of patients remaining parasitaemic at 24, 48 and 72 hours was documented in 115 (28%), 167 (40%) and 153 (37%) treatment arms, respectively. Excluding resistance studies in Cambodia, the median proportion of patients still parasitaemic was 53.8% [range 3–95, IQR=30.5-69.2] on Day 1, 6% [range 0–65.9, IQR=2-11.5] on Day 2 and 0 [range 0–12.6, IQR=0-2] on Day 3. Comparing studies from 2000 to 2005 and 2006 to 2011, the median proportion of patients reported to remain parasitaemic at 72 hours decreased in Africa (1.2% *vs* 0%, p=0.007), but increased in Asia (0.4% *vs* 3.9%, p=0.076). In 95% of studies the proportion of patients with peripheral parasitaemia was less than 6% at 72 hours.

**Conclusions:**

These results highlight the normal distribution of early parasitological responses following ACT, and the influence that heterogeneity in study design, host and parasite factors have in confounding a surveillance system based on Day 3 parasite positivity. Greater understanding of factors influencing parasite clearance is crucial, but will require analysis of pooled data from individual patient records.

## Background

Artemisinin-based combination therapy (ACT) is currently the first-line treatment for uncomplicated *Plasmodium falciparum* malaria recommended by the World Health Organization (WHO) [1]. Substantial decrease in malaria attributable morbidity and mortality in some endemic areas has been credited to the intensive deployment of highly effective ACT treatment regimens and the widespread use of long-lasting insecticide-treated bed nets (LLINs) [2]. However the continued success of these malaria control programmes is under serious threat from the emergence of artemisinin-resistant strains of *P. falciparum* reported from the Thai-Cambodian border [3-5] and more recently from the Thai-Myanmar border [6].

The artemisinin derivatives are the most potent anti-malarial drugs, with a broad-stage specificity against young ring-stage parasites, trophozoites and early gametocytes stages. The rationale behind ACT relies on the high potency of artemisinin affecting a rapid reduction in peripheral parasitaemia, thus reducing the parasite biomass remaining to be eliminated by the longer-acting partner drug [7]. Although the overall efficacy of ACT is dependent on both drugs, the initial parasite clearance rates are predominantly a function of artemisinin activity. The hallmark of artemisinin resistance comes from clinical observations of a delayed early clearance of detectable parasitaemia in the peripheral blood film, associated with an increase in parasite gametocyte and thus transmissibility [3,4,6].

Surveillance of artemisinin resistance and delineation of the boundaries of spread is critical if appropriate public health measures are to be taken to contain or at least delay the evolution of the process. As yet there are no validated molecular markers of artemisinin resistance, and current *in vitro* assays have proven insensitive at identifying parasites associated with a clinical resistance phenotype [8]. For this reason, surveillance strategies have been devised to define resistance from the early clearance of peripheral parasitaemia. A variety of parameters have been developed which vary in complexity as well as sensitivity. Frequent sampling of the parasite response and derivation of the rate of reduction has been proposed as a reliable method for defining the parasite susceptibility to artemisinin derivatives [9]. However such tests are logistically more difficult to conduct than the current standard *in vivo* efficacy assessment [10], and require appropriately designed prospective application [11].

An alternative approach advocates the use of the proportion of patients with detectable parasitaemia in a blood smear taken on Day 3, 72 hours after starting anti-malarial treatment [12]. When more than 10% of patients have a detectable peripheral parasitaemia on Day 3 this is considered to be indicative of suspected artemisinin resistance. Such a definition is relatively simple to implement and can be applied retrospectively from completed clinical trials adhering to established WHO guidelines on monitoring anti-malarial drug resistance, providing a minimum of 50 patients have been enrolled in the study [13]. However this simple approach is vulnerable to a number of important factors that may confound its interpretation.

In the current study the utility of applying a 10% parasite positivity threshold on Day 3 as a marker of artemisinin resistance, was reviewed from all available published data from the last 12 years involving artemisinin derivatives. The objectives of the study were to review the information currently available on parasitological measures of artemisinin-containing clinical trials and to document early parasitological responses to treatment.

## Methods

### Building the reference library

**Figure 1:**
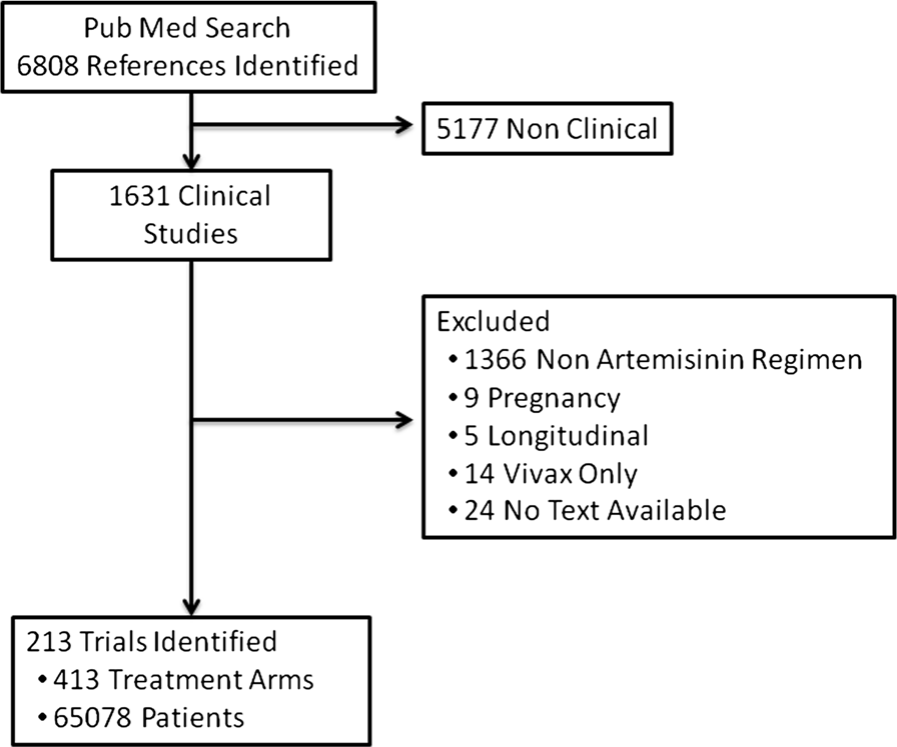
Study profile.

A systematic search of the literature was conducted to identify all published antimalarial clinical efficacy studies conducted since 1960. The key terms for the search are presented in the additional files (see Additional file 1). References were manually checked to confirm prospective clinical trials; these are available on the WWARN website [14]. From this reference library all studies assessing regimens containing an artemisinin derivative between January 2000 and December 2011 were identified (see Additional file 2). Studies involving only non-artemisinin regimen, pregnancy trials, longitudinal studies, and studies of patients without *P. falciparum* infection were excluded. The study profile generated by the search algorithm is summarized in Figure 1.

### Variables extracted

Each publication was reviewed and trial data extracted to a standard record form. Variables included details on the study location(s), randomization process, duration of follow up, baseline characteristics, treatment given (including dose and duration of artemisinin derivatives and partner drugs), and parasite positivity rates (PPR) on Day 1, 2 and 3 (see Additional files 3, 4 and 5).

### Statistical analysis

Data were extracted and entered into Microsoft Office Access™. All analyses were conducted using Stata software, version 11.2 (StataCorp). The Mann–Whitney U test or Kruskal-Wallis method were used for nonparametric comparisons, and for categorical variables, proportions were examined using Chi squared test with Yates' correction or by Fisher's exact test. Logistic regression model with a study random effect on individual patient data was used to determine the effect of study characteristics (year, location, partner drug, artemisinin derivative, dose of artemisinin derivative) and patient characteristics (age and parasite density) on parasite positivity rates at 24, 48 and 72 hours. The effect of individual patient characteristics was investigated using summary statistics reported in the publications and limitations of this methodology have been previously reported [15]. To maximize available data when the number of patients evaluated for parasitaemia was not stated in the text, it was assumed that all patients in that study arm had been evaluated at that time point. In the meta-analysis, proportions were transformed using the Freeman-Tukey variant of the arcsine square root transformation and random effects model using the method of DerSimonian & Laird, with the estimates of heterogeneity derived from the Mantel-Haenszel model [16,17].

## Results

**Figure 2:**
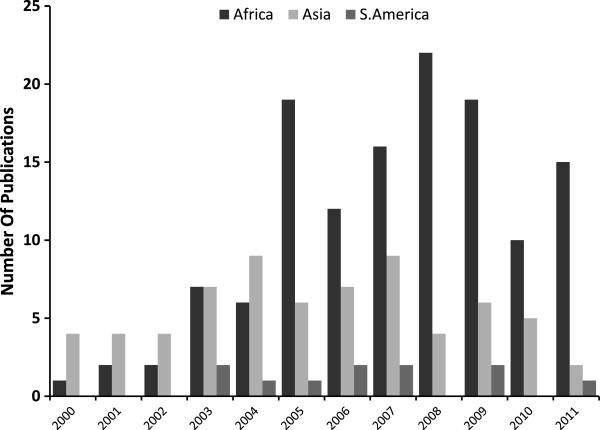
Temporal trends in publication of anti-malarial clinical trials with an artemisinin-based combination therapy.

Between January 2000 and December 2011, 213 clinical trials were identified in which a total of 65,078 patients with uncomplicated falciparum malaria were enrolled into 413 treatment arms containing an artemisinin derivative (Figure 1). The median sample size per treatment arm was 110 (range 10–1,475), with most of the data derived from comparative drug trials (189, 89%), of which 177 (83%) were randomized trials. The remaining 24 (11%) studies were single-arm studies. The number of trials published annually increased during the study period from five publications in 2000 to a peak of 28 in 2009, although numbers declined thereafter (Figure 2). Overall 37,192 (57%) patients had *P. falciparum* mono-infection and 7,908 (12%) had mixed infections on admission. The remaining 19,978 patients were enrolled in studies in which parasite species was not stated; these were mostly conducted in Africa. Follow up was continued for 28 days in 134 (63%) studies, with 50 (24%) extending to 42 days, and 10 (5%) up to 63 days. The proportion of trials with at least 42 days of follow up rose from 20% (15/74) in the period between 2000 and 2005 to 36% (49/138) between 2006 and 2011; p=0.027. The most frequent treatment regimens reported were: artemether-lumefantrine (AL, in 96 treatment arms), artesunate-mefloquine (AS+MQ, N=75), artesunate-amodiaquine (AS+AQ, N=74), artesunate-sulphadoxine-pyrimethamine (AS+SP, N=43), and dihydroartemisinin-piperaquine (DHA+PIP, N=40). In 26 treatment arms, patients received monotherapy only, including either artesunate (N=17), dihydroartemisinin (N=1), artemisinin (N=3), beta-artemether (N=2) and arterolane (N=3). Treatment was supervised completely in 362 (90%) and partially in 30 (7.5%) of the 402 studies in which it this was documented.

**Figure 3:**
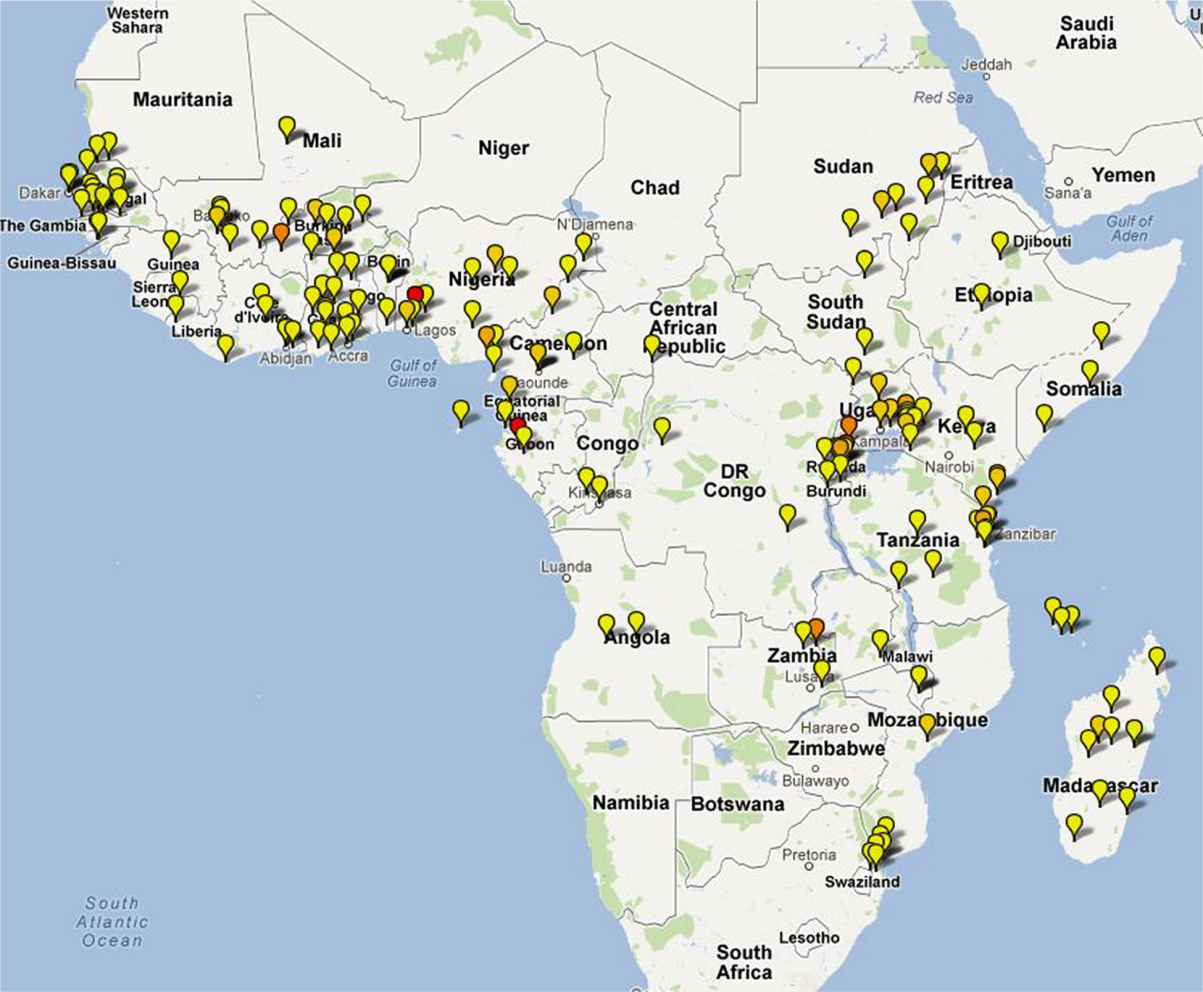
**Map depicting location of artemisinin-based combination therapy clinical studies conducted in Africa since 2000.** Coloured icons denote number of studies at each site (yellow = 1; light orange = 2; dark orange = 3; red = >4).

**Figure 4:**
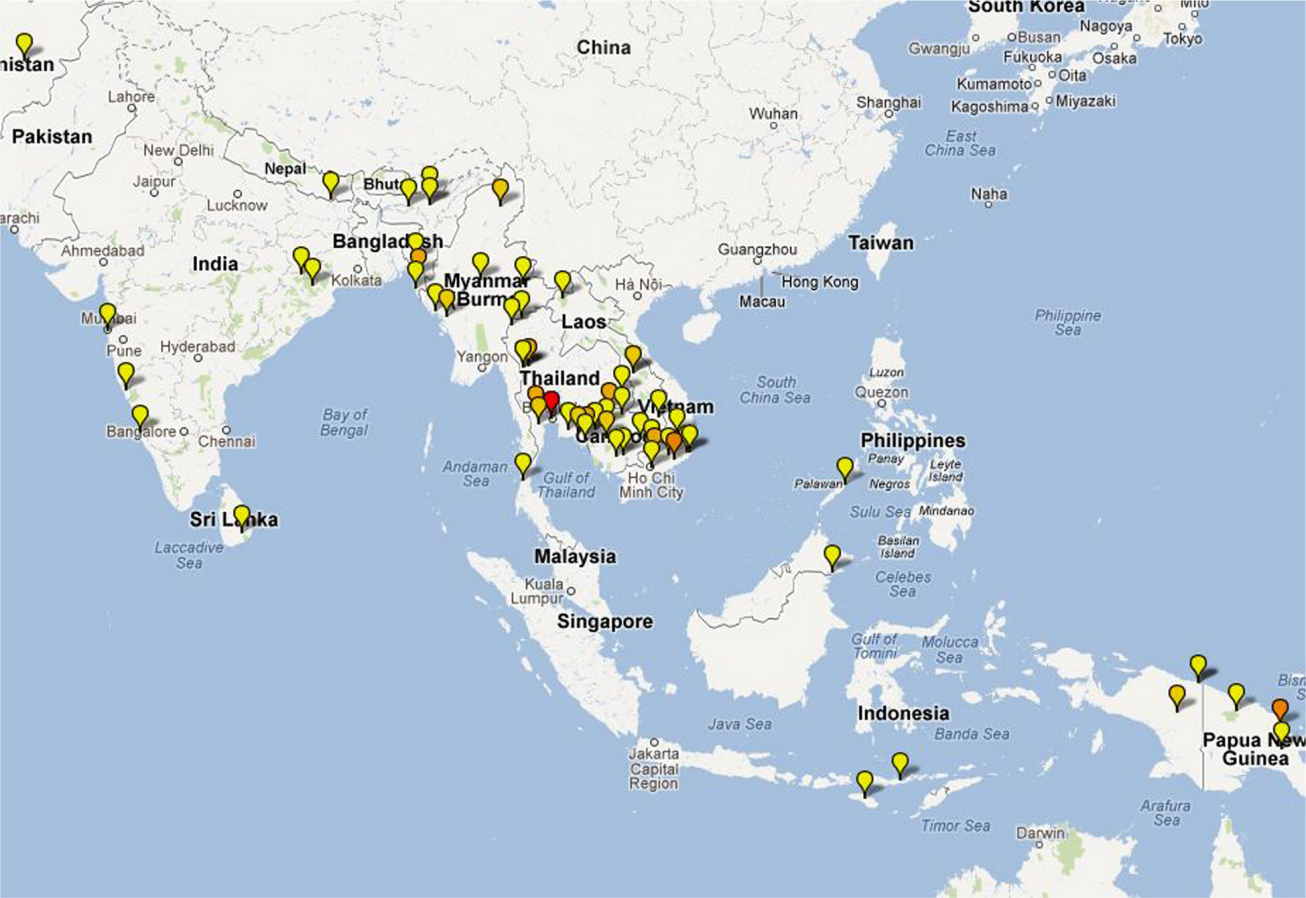
**Map depicting location of artemisinin-based combination therapy clinical studies conducted in Asia since 2000.** Coloured icons denote number of studies at each site (yellow = 1; light orange = 2; dark orange = 3; red = >4).

**Figure 5:**
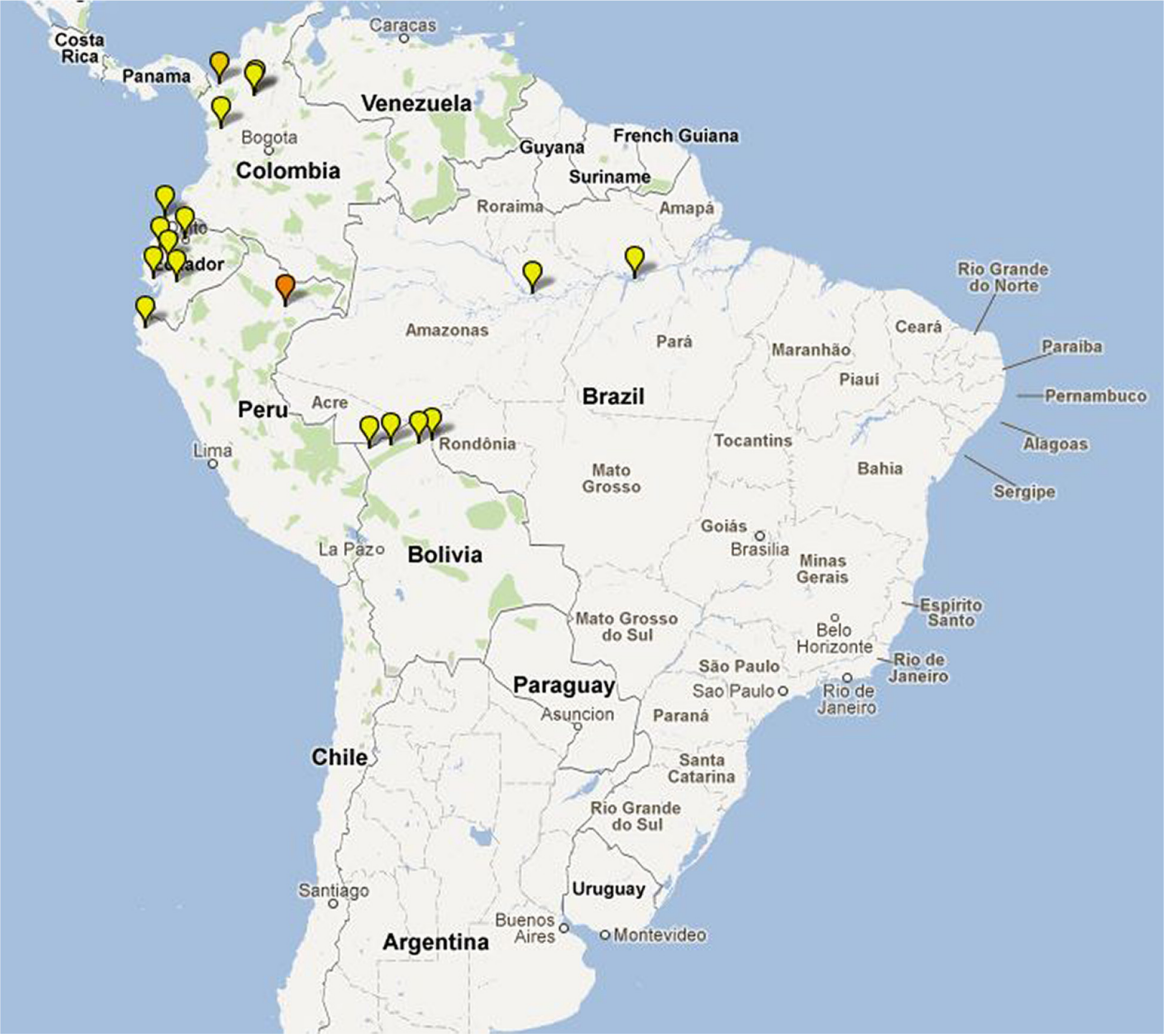
**Map depicting location of artemisinin-based combination therapy clinical studies conducted in Latin America since 2000.** Coloured icons denote number of studies at each site (yellow = 1; light orange = 2; dark orange = 3; red = >4).

The majority of studies came from Africa, which contributed 131 (62%) studies and 43,982 (68%) patients. There were 67 (31%) trials in Asia recruiting 18,083 (28%) patients and 11 studies from South America recruiting 1,295 (2%) patients. Locations of these study sites are presented in Figures 3, 4 and 5. A total of 21 multicentric trials (involving at least two countries) were reported, 17 of which were conducted in Africa, three trials in Asia and the other in South America. Two studies included sites in both continents. African trials were more likely to recruit children (<14 years), with only 3% (4/131) of trials enrolling adults; the median age reported being four years (range 0.6-39.5). In contrast, 94% (63/67) of Asian studies enrolled adults with a median age of 24 years (range 2.9-38). The median baseline parasitaemia reported was 22,905 μl^-1^ (range 2,700-105,426) in African trials, compared to 8,986 μl^-1^ (range 536–65,299) in Asia (see Additional file 4).

In 11 South American trials, the median (range) baseline parasitaemia was 4,445 μl^-1^ (range 2,838-7,309) and the median age at enrolment was 25.9 years (range 6.7-36). Of the 1,295 patients recruited in South America, 59% (762) received AS+MQ in 10 treatment arms, 20% (262) were allocated to DHA+PIP, 12% (151) to AS+SP and the remainder were treated by AL, AS+AQ or artesunate rectal capsule.

### Parasitologic response during the first three days

The early parasitological response to treatment was reported in 60% (128/213) of studies, with the parasite positivity stated (or derived) at Day 1 in 115 (28%) treatment arms, at Day 2 in 167 (40%) treatment arms and at Day 3 in 153 (37%) treatments arms. Most studies (53% 113/213) assessed blood smear once daily, but in 27% (58/213) of studies examination was more frequent (four to 12 hourly). In 29 (14%) studies parasitaemia was reported less than once daily and the remainder (13 studies) did not specify intervals of blood slide taken in the methodology.

The proportion of patients with parasitaemia on Days 1, 2 and 3 and the median parasite positivity rates (PPR) derived from studies are presented in Table 1, with individual PPRs at Day 2 presented in Additional files 3 and 5).

**Table 1:** Parasite positivity according to day of follow up and continent

	**Proportion of patients with patent parasitaemia**		**Parasite positivity rate**		
			**Number of study arms**	**Median %**	**[Range,IQR]**
**Africa**					
Day 1	53.2%	(6,694/12,576)	76	48.9%	[Range: 2.6-94.4 IQR: 26.6-66.4]
Day 2	8.3%	(1,575/19,044)	111	6.1%	[Range: 0–65.9, IQR: 1.6-11.2 ]
	7.9%	(1,456/18,466)	107	6.0%	[Range: 0–65.9, IQR: 1.6-11.0 ]
Day 3	1.0%	(238/18,593)	115	0%	[Range: 0–36.5, IQR: 0–1.2 ]
	1.0%	(176/17,223)	107	0%	[Range: 0–10.0, IQR: 0–1.2 ]
**Asia**					
Day 1	59.0%	(2,794/4,733)	30	59.9%	[Range: 0—84.3, IQR: 37.7-74.0]
	59.5%	(2,793/4,691)	28	61.6%	[Range: 16.4—84.3, IQR: 41.1-74.0]
Day 2	12.2%	(808/6,608)	45	8.9%	[Range: 0–72.5, IQR: 3.3-20.8]
Day 3	3.8%	(247/6,651)	45	1.1%	[Range: 0–55.0, IQR: 0–5.3]
	3.8%	(243/6,399)	41	1.7%	[Range: 0–55.0, IQR: 0–5.6]
**South America**					
Day 1	39.6%	(237/598)	5	48.0%	[Range: 32.3-75.0, IQR=41.5-48.0]
Day 2	6.0%	(43/719)	7	4.0%	[Range: 0–21.4, IQR=1.1-17.9]
Day 3	1.0%	(10/1044)	11	0%	[Range: 0–8.6, IQR=0-2.1]

First row in each day category shows all reported results. The second row (if present) shows the results after exclusion of study arms with rectal artesunate and with short (<1, 2 or 3 days, respectively) treatment with artemisinin derivatives.

### Factors influencing early parasite responses

Parasite positivity rate at Day 1 (PPRday1) and Day 2 (PPRday2) did not differ between Asia and Africa, but by Day 3 (PPRday3) patients from Asian studies were at significantly greater risk of remaining parasitaemic (Odds Ratio OR = 3.58 [95% CI 1.15 – 10.13]; p=0.027).

The proportion of patients with detectable parasitaemia depended significantly on the number of days in which an artemisinin derivative treatment had been administered. In Africa the OR [95% CI] for PPRday2 was 2.72 [1.90-3.92] for regimens with less than two days of artemisinin derivative therapy (four arms with 588 patients) and was 6.19 [3.43-11.17] for PPRday3 for treatments with shorter than three days (nine arms with 1,370 patients) compared to regimens with longer courses of artemisinin derivatives. In Asia, all treatments included at least three days of artemisinin derivatives, except for 182 patients in two study arms who received only two days. Further analyses of PPRday2 were restricted to patients who received at least two days of artemisinin derivatives and of PPRday3 to patients who received at least three days of artemisinin derivatives.

In African studies, the median age of the patient was associated with a reduced risk of parasitaemia at Day 1 (OR 0.97 [95% CI 0.95 - 0.99]), but not on subsequent days (Table 2). Increasing parasitaemia at presentation was associated with a higher PPR on Day 1 (OR 1.74 [95% CI 1.26 -2.39]), Day 2 (OR 1.74 [95% CI 1.25 - 2.42]) and Day 3 (OR 2.68 [95% CI 1.00-7.18]), for unit exponential increase). There was also a significant difference in the parasite positivity rates between treatment regimens: patients treated with AL were more likely to be parasitaemic than those treated with DHA+PIP at both 24 hours (OR = 1.50 [95% CI 1.19 - 1.88]) and 48 hours (OR 1.70 [95% CI 1.21 -2.40]). Although a similar difference was also noted for three-day regimens of AS+SP and AS+AQ, this was only apparent at 48 hours (OR 2.17 [95% CI 1.33-3.54] and OR 2.05 [95% CI 1.39-3.03], respectively).

**Table 2:** Univariate analysis of factors associated with early parasite response in Africa

	**Day 1**		**Day 2**		**Day 3**	
	**OR (95% CI)**	**P-value**	**OR (95% CI)**	**P-value**	**OR (95% CI)**	**P-value**
**Age**	0.97 (0.95-0.99)	0.016	1.01 (0.97-1.04)	0.767	1.04 (0.96-1.12)	0.354
**Log**_**e **_**parasitaemia**	1.74 (1.26-2.39)	0.001	1.74 (1.25-2.42)	0.001	2.68 (1.00-7.18)	0.050
**Year**	0.88 (0.78-1.00)	0.051	0.89 (0.78-1.02)	0.087	0.79 (0.64-0.99)	0.039
**Artemisinin derivative**		<0.001		0.002		0.141
**Artemether**	1.00		1.00		1.00	
**Artemisinin**	0.87 (0.41-1.86)	0.718	NA		NA	
**Artesunate**	0.83 (0.72-0.95)	0.009	1.06 (0.84-1.34)	0.617	1.16 (0.56-2.41)	0.692
**DHA**	0.65 (0.51-0.83)	<0.001	0.57 (0.40-0.80)	0.001	0.21 (0.02-1.79)	0.154
**Treatment**		0.002		0.004		0.494
**DHA+PIP**	1.00		1.00		1.00	
**AL**	1.50 (1.19-1.88)	0.001	1.70 (1.21-2.40)	0.002	4.70 (0.55-40.03)	0.157
**AS+MQ**	1.34 (0.86-2.08)	0.19	2.25 (0.98-5.14)	0.055	6.03 (0.43-84.46)	0.182
**AS+SP**	1.08 (0.48-2.43)	0.851	2.17 (1.33-3.54)	0.002	7.60 (0.86-66.87)	0.067
**AS+AQ**	1.30 (0.98-1.73)	0.073	2.05 (1.39-3.03)	<0.001	5.99 (0.74-48.64)	0.094
**AS alone**	0.73 (0.16-3.29)	0.685	0.76 (0.29-2.01)	0.583	ND^1^	

Only patients who received artemisinin derivative until Day 1 (2 or 3) are included for analysis of Day 1 (2 or 3). Study arms with rectal artesunate were excluded. Positivity rate equal to 0% on Day 2 was carried forward to Day 3 if Day 3 rate was not reported.

ND= There were no patients available with detectable parasitaemia in this category so the OR could not be estimated. P-value = 1.000 for comparison with DHA+PIP treatment (Fisher’s exact test). For the purpose of logistic model this treatment group was merged with all other treatments (not included in the listed categories).

In Asian studies, there were no significant differences in initial parasite clearance between treatment regimens at 48 or 72 hours (Table 3). However, increasing baseline parasitaemia was associated with higher PPRday1 (OR 2.42 [95% CI 1.58-3.70]), PPRday2 (OR 3.11 [95% CI 1.86-5.20]) and PPRday3 (OR 8.20 [95% CI 3.97-16.97] per unit exponential increase). This relationship remained significant at 48 hours but not at 72 hours, when studies from Cambodia, where artemisinin resistance has been confirmed, were excluded.

**Table 3:** Univariate risk factors for early parasitaemia positivity rates in Asia

	**Day 1**		**Day 2**		**Day 3**	
	**OR (95% CI)**	**P-value**	**OR (95% CI)**	**P-value**	**OR (95% CI)**	**P-value**
**Age**	1.03 (0.95-1.12)	0.474	0.96 (0.89-1.02)	0.185	0.85 (0.79-0.92)	<0.001
**Log**_**e **_**parasitaemia**	2.42 (1.58-3.70)	<0.001	3.11 (1.86-5.20)	<0.001	8.20 (3.97-16.97)	<0.001
**Year**	0.98 (0.82-1.16)	0.789	0.99 (0.76-1.30)	0.959	1.43 (1.07-1.91)	0.015
**Artemisinin derivative**		<0.001		0.904		0.231
**Artemether**	1.00		1.00		1.00	
**Artemisinin**	ND		1.45 (0.25-8.43)	0.676	ND	
**Artesunate**	0.83 (0.68-1.01)	0.058	1.06 (0.72-1.56)	0.784	3.85 (0.73-20.30)	0.113
**DHA**	0.50 (0.40-0.62)	<0.001	0.97 (0.63-1.50)	0.904	3.39 (0.59-19.59)	0.173
**Treatment**		<0.001		0.778		0.592
**DHA+PIP**	1.00		1.00		1.00	
**AL**	2.04 (1.62-2.57)	<0.001	1.02 (0.66-1.57)	0.934	0.29 (0.05-1.67)	0.167
**AS+MQ**	1.64 (1.36-1.98)	<0.001	1.09 (0.85-1.41)	0.487	1.16 (0.65-2.06)	0.620
**AS+SP**	0.46 (0.12-1.86)	0.279	0.09 (0.003-2.68)	0.166	ND	
**AS+AQ**	0.66 (0.11-4.04)	0.654	0.45 (0.03-5.94)	0.547	2.28 (0.16-32.22)	0.543
**AS alone**	ND		1.25 (0.64-2.45)	0.512	1.45 (0.59-3.54)	0.414

Only patients who received artemisinin derivative until Day 1 (2 or 3) are included for analysis of PPRday1 (2 or 3). Study arms with rectal artesunate are excluded. Positivity rate equal to 0% on Day 2 was carried forward to Day 3 if Day 3 rate was not reported.

ND= There were no patient data available or patients with detectable parasitaemia in this category so the OR could not be estimated. P-value = 0.623 for comparison with DHA+PIP treatment (Fisher’s exact test). For the purpose of logistic model this treatment group was merged with all other treatments (not included in the listed categories).

In African studies, univariate factors were confirmed in multivariate analysis of parasitaemia at Day 1 and Day 2 (Table 4). After correcting for age, baseline parasitaemia and year, patients treated with AL (adjusted OR (AOR) 1.61 [95% CI 1.14-2.28]), AS+SP (AOR 1.91 [95% CI 1.16-3.14]) and AS+AQ (AOR 1.84 [95% CI 1.23-2.74]) were significantly more likely to be parasitaemic at Day 2 compared to those receiving DHA+PIP. At Day 3, there were no significant differences between treatments.

**Table 4:** Multivariable risk factors for early parasitaemia positivity rates in Africa

	**Day 1**		**Day 2**		**Day 3**	
**Variable**	**AOR (95% CI)**	**P-value**	**AOR (95% CI)**	**P-value**	**AOR (95% CI)**	**P-value**
**Age**	0.99 (0.97-1.02)	0.689	1.02 (0.98-1.06)	0.368	1.04 (0.97-1.12)	0.252
**Log**_**e **_**parasitaemia**	1.76 (1.18-2.62)	0.005	1.59 (1.16-2.18)	0.004	3.01 (1.18-7.64)	0.021
**Year**	0.84 (0.73-0.96)	0.012	0.92 (0.79-1.07)	0.270	0.78 (0.62-0.97)	0.026
**Treatment**						
**DHA+PIP**	1.00		1.00		1.00	
**AL**	1.32 (1.02-1.71)	0.033	1.61 (1.14-2.28)	0.007	5.03 (0.58 – 43.52)	0.143
**AS+MQ**	1.22 (0.77-1.91)	0.398	2.04 (0.84-4.94)	0.115	9.45(0.61-146.06.76)	0.108
**AS+SP**	0.74 (0.32-1.74)	0.495	1.91 (1.16-3.14)	0.011	4.89 (0.50-47.34)	0.171
**AS+AQ**	1.06 (0.76-1.46)	0.740	1.84 (1.23-2.74)	0.003	4.98 (0.59-42.07)	0.141
**AS alone**	0.55(0.12-2.50)	0.439	0.74 (0.28-1.97)	0.548	ND	

Only patients who received artemisinin derivative until Day 1 (2 or 3) are included for analysis of PPRday1 (2 or 3). Study arms with rectal artesunate are excluded. Positivity rate equal to 0% on Day 2 was carried forward to Day 3 if Day 3 rate was not reported.

ND= There were no patient data available or patients with detectable parasitaemia in this category so the OR could not be estimated. For the purpose of logistic model this treatment group was merged with all other treatments (not included in the listed categories).

In the multivariate analysis of the Asian studies, baseline parasitaemia, age and treatment regimen also remained significantly associated with delayed parasite clearance (Table 5). After correcting for confounding factors, treatment with AL (AOR = 1.81 [95% CI 1.42-2.31]) or AS+MQ (AOR = 1.40 [1.12-1.76]) was associated with higher PPRday1 than treatment with DHA+PIP. Patients treated with AL had higher PPRday1 than either AS+MQ (AOR=1.29 [1.01-1.65]) or AS+AQ (AOR =2.72 [1.15-6.44]. At Day 2 and Day 3 the only factor independently associated with parasite clearance was baseline parasitaemia.

**Table 5:** Multivariate logistic regression model for early parasite response in Asia

	**Day 1**	
**Variable**	**AOR (95% CI)**	**P-value**
**Age**	1.08 (1.02-1.14)	0.008
**Log**_**e **_**parasitaemia**	2.65 (1.67-4.21)	<0.001
**Year**	0.90 (0.81-0.99)	0.039
**Treatment**		
**DHA+PIP**	1.00	-
**AL**	1.81 (1.42-2.31)	<0.001
**AS+MQ**	1.40 (1.12-1.76)	0.003
**AS+SP**	2.23 (0.67-7.42)	0.192
**AS+AQ**	0.66 (0.28-1.56)	0.342
**AS alone**	ND	

Only patients who received artemisinin derivative until Day 1 are included in the analysis of PPR1. Study arms with rectal artesunate are excluded.

ND= There were no patient data available.

### Temporal trends

In the studies conducted in Asia, there was a temporal trend for an increased risk of parasite positivity at Day 1 (AOR = 0.90 [95% CI 0.81-0.99] per year), but not at other time points. In African studies, the reverse was observed, with a linear trend for decreased positivity at all days, but only statistically significant on Day 1 and Day 3 (AOR= 0.84 [95% CI 0.73-0.96]; AOR = 0.78 [95% CI 0.62-0.97] per year, respectively).

Temporal trends in parasitological response were assessed in studies conducted from 2000 to 2005 and compared with 2006 to 2011. In African studies, the median reported proportion of patients remaining parasitaemic during follow up decreased at Day 1 (64% to 43%), Day 2 (10% to 4.6%) and Day 3 (1.2% to 0%). However these changes, adjusted for confounding factors, were not statistically significant in the logistic regression model. In contrast, in Asian studies the median proportion of patients parasitaemic at Day 3 rose from 0.4% in 2000 to 2005 to 6.5% in 2006 to 2011. However after adjusting for confounding factors, and exclusion of Cambodian studies, these temporal trends were no longer statistically significant.

### Identifying areas of delayed parasite clearance

**Figure 6:**
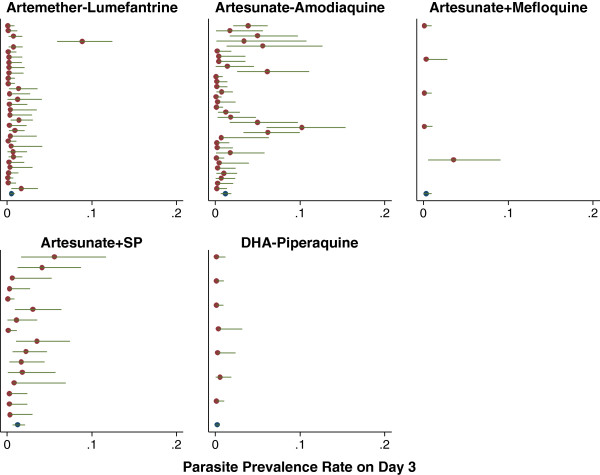
**Forest plots of Day 3 parasite positivity rates in Africa.** Estimates and 95% CI are shown by treatment, sorted by year in descending order (most recent first). Heterogeneity between studies I^2^: AL = 63.4%; AS+AQ = 75.5%; AS+MQ = 35.1%; AS+SP = 52.1%; PIP+DHA = 0.0%.

**Figure 7:**
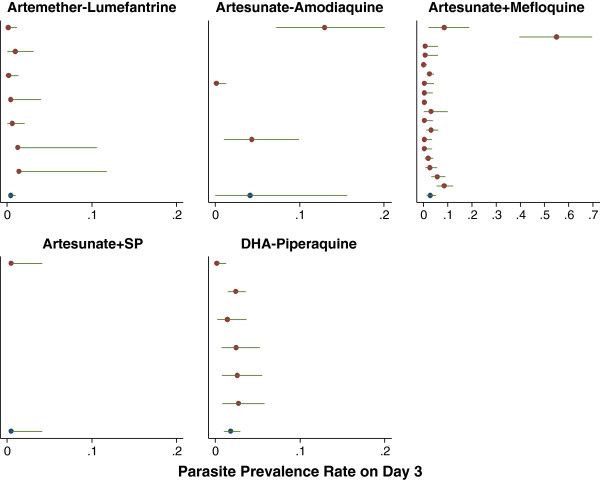
**Forest plots of Day 3 parasite positivity rates in Asia.** Estimates and 95% CI are shown by treatment, sorted by year in descending order (most recent first). Heterogeneity between studies I^2^: AL = 0.0%; AS+AQ = 93.0%; AS+MQ = 88.0%; AS+SP = NA; PIP+DHA = 44.9%.

The parasite prevalence rates and 95% confidence intervals are presented in the forest plots at Day 3 (Figures 6 and 7) and at Day 2 (Additional files 6 and 7). Overall the median proportion of patients remaining parasitaemic after three-day regimens was 53.8% [range 3–95, IQR=30.5-69.2] at Day 1, 6.7% [range 0–73, IQR=2-12.9] at Day 2 and 0.5% [range 0–55, IQR=0-2.2] at Day 3. When studies from Cambodia were excluded, the corresponding proportions were 53.8% [range 3–95, IQR=30.5-69.2], 6.0% [range 0–65.9, IQR=2-11.5] and 0 [range 0–12.6, IQR=0-2]. In 95% of studies the proportion of patients with peripheral parasitaemia was <84% at Day 1, <34.5% at Day 2 and <6% at Day 3. In comparison, the studies from Western Cambodia reported median parasite positivity rates of 60% [range 48–73] at Day 2 and 48% [range 22–55] at Day 3.

Among 153 treatment arms reporting parasite positivity rate at Day 3, nine (5.9%) recorded a PPRday3 of 10% or higher. Of them, six treatment regimens were evaluated in Western Cambodia and one study was of a single dose of artesunate (see Additional file 8). In the remaining two studies [18,19] patients were treated with a three-day AS+AQ in Senegal and Indonesia and with PPRday3 documented as 10% (95%CI =6.3-15.6%) and 12.6% (95% CI=7.5-20.4%) respectively.

## Discussion

The emergence and spread of resistance to anti-malarial drugs has proven to be a crucial factor undermining malaria control programmes and global aspirations for the elimination of malaria. Anti-malarial resistance has emerged to nearly all established anti-malarial compounds and is considered as inevitable for any drugs that are in common use and deployed widely. The consequences of anti-malarial drug resistance are huge and are associated with greater malaria attributable morbidity and mortality. The use of ACT was designed to combat anti-malarial drug resistance, increasing the treatment efficacy against even the most multidrug resistant isolates, reducing the transmission potential of the parasite and preventing the emergence of *de novo* resistance. Over the last decade more than 80 countries have adopted ACT as a key component of national anti-malarial guidelines. However, this strategy is now under threat with reports of declining artemisinin efficacy against *P. falciparum* infection. The first documentation of declining susceptibility came from Pailin in western Cambodia, and has now been confirmed on the western border of Thailand, and Vietnam [3,4,6]. The threat of an escalation to high-grade artemisinin resistance spreading throughout the malaria-endemic world is alarming and demands immediate action to contain potential hotspots and retard the spread of resistant parasites to new areas. To counter this threat, WHO launched a coordinated plan outlined in the Global Plan for Artemisinin Resistance Containment (GPARC), which proposed an international commitment to both accelerate malaria control efforts and implement a number of additional activities [13].

The hallmark of declining artemisinin efficacy is a delay in the early parasite clearance times. Prolonged parasite clearance increases the risk of gametocyte carriage and thus transmission potential, as well as increasing parasite exposure to the partner drug and thus the risk of selecting for further drug-resistant isolates. The assessment of therapeutic clinical response, particularly the early parasite positivity rate has been identified as a crucial part of drug efficacy monitoring for artemisinin resistance, but its interpretation is vulnerable to a number of confounding factors [20,21]. Novel approaches to quantify the therapeutic efficacy have attempted to overcome this by frequent blood film examination (at least eight-hourly) and analysis of the slope of the parasitaemia clearance profiles [9]. Although such studies are now being conducted prospectively by research groups, their application is a significant change from current *in vivo* protocols for uncomplicated malaria which have derived the early parasite clearance mostly from once-daily sampling. Simplified sampling strategies, although less precise, have the advantage of being adopted across a wider geographical area and logistical framework. Furthermore, a system that is compatible with current protocols permits the analysis of data that has already been gathered, to be re-examined to identify potential hot spots of emerging artemisinin resistance. Monitoring of the Day 3 parasite positivity rate has been proposed as a simple, robust surveillance tool, with the rationale that this crude test could identify sites warranting further more detailed clinical investigation [12]. However, the sensitivity, specificity and ultimate utility of such an approach will depend upon the threshold chosen to signal a poor outcome and acknowledgement for the host, parasite and drug factors that can either mask declining susceptibility to artemisinin or give rise to false alarm.

The purpose of the current study was to review the recent literature of clinical studies of artemisinin derivatives in order to quantify the distribution of parasite clearance times and the cofactors underlying these. Important variations are apparent in the design and methodology of ACT clinical drug trials, indicative on the rationale of the study, local epidemiology, as well as logistical and funding constraints. In particular microcopy protocols can vary considerably, and although standardised quantification of thick and thin films are crucial details of their underlying methodologies are rarely presented. Furthermore there were significant differences in both the age of inclusion and baseline parasitaemia, both of which are critical to the speed of parasite clearance [12].

Although almost two thirds of studies reviewed in this analysis, reported details on the early parasitological response, these were reported inconsistently with only 40% providing parasite positivity rates on Day 2 and 37% on Day 3. Furthermore, 13% (n=28) of these data points were estimated from figures presented to depict parasitological response rather than the text itself. From the data available, 91% of patients had cleared their parasitaemia within two days and 97.9% within three days, however there was marked heterogeneity in clearance rates between studies and regions (highlighted in Figures 6 and 7). Several important confounding factors were identified. As expected, baseline parasitaemia was major risk factor for patent parasitaemia particularly on Day 3. Parasite positivity rates over the first day of treatment were also significantly higher in children than adults, although this was only apparent in Asian studies where a greater spectrum of age groups was recruited. After controlling for these factors, parasite positivity rates varied considerably with the treatment regimen administered, short course treatments being associated with two- to six-fold higher rates of parasitaemia at Day 2 and Day 3. Even when the analysis was restricted to three-day regimens, the differences in parasite positivity rates remained, with DHA-piperaquine associated with significantly greater clearance than AL and AS+MQ (see Tables 2 and 3).

Over the last decade the analysis of temporal trends in parasite clearance reveal a significant increase in the proportion of patients remaining parasitaemic at Day 3 in Asia, however this was mostly accounted for by the recent Cambodian studies which were clear outliers. Outside of Cambodia no significant trend was apparent. Conversely in Africa, the parasite positivity rate was noted to decrease over the decade at each day of follow up, although this was mostly attributable to variation in the age of the patients and baseline parasitaemia and was not apparent in multivariable analysis. After excluding Cambodian studies, thresholds expected to be achieved in 95% of clinical studies could be estimated as a parasite positivity rates less than 84% at Day 1, less than 35% at Day 2 and less than 6% at Day 3. The current threshold for alerting for a potential decline with artemisinin resistance has been proposed as a PPR on Day 3 of greater than 10%, well above the 95^th^ percentile. Using this definition, nine outliers were identified, six of which were from the Pailin region, the epicentre for emerging artemisinin resistance in Cambodia and one of which was a short-course regimen. Of the remaining two studies, one by Asih *et al.* from Indonesia conducted in 2005 to 2006, combined PCR positivity with microscopy, which may have accounted for higher than expected proportion of patients parasitaemic on Day 3 [19]. The remaining trial arm in a study by Adjuik *et al.* was conducted in Senegal, although the Day 3 value was derived from a figure rather than actual data [18], (see Additional file 8).

The present analysis relies on aggregated data collected mostly from studies with once daily sampling. The specificity of the 10% Day 3 PPR cut off in detecting areas of declining artemisinin efficacy could be estimated at 67%, assuming that the two studies from Senegal and Indonesia was false positives. However it was not possible to determine the sensitivity of this clinical marker particularly for detecting early indications of emerging resistance. From the data presented here and previous analyses it is clear that the interpretation of simple measures of parasite clearance are highly vulnerable to host, parasite and drug factors, all of which vary significantly with heterogeneous study designs. Standardization of methodology, unified approaches for reporting, and the application of more stringent inclusion and exclusion enrolment criteria may go some way to controlling for these. The WHO malaria guidelines on monitoring *in vivo* drug resistance are regularly reviewed and updated and are crucial in encouraging a standardized approach to global drug resistance monitoring [10]. Complementary pooled analyses of individual level data allow multivariate analysis, which increase the power to identify locations of outlying studies that defy what has historically been an extremely rapid parasitological response. Current efforts by the WorldWide Antimalarial Resistance Network (WWARN) are directed at facilitating such approaches, using subject-level data and standardizing analytical outputs, ensuring that relevant and validated data can be explored controlling for confounding factors. Such endeavours are of critical importance in building strong collaborative commitments from the global malaria community to tackle a major public health threat.

## Competing interests

The authors declare that they have no competing interests.

## Authors’ contributions

PG, RP and KS designed the study. DD reviewed literatures and extracted data. DD, KS and RP analysed the data. DD drafted the manuscript. RP, DB, KS, and PG reviewed and edited the manuscript. All authors read and approved the final manuscript.

## Supplementary Material

Additional file 1 PubMed search terms. Click here for file

Additional file 2 Study references included in the analysis. Click here for file

Additional file 3 Extracted Data. Click here for file

Additional file 4 **Study design of trials included in the analysis.** Parasitaemia presented as geometric mean or mean, unless marked with * in which case median. Age presented as mean, unless marked with * in which case median. Click here for file

Additional file 5 **Parasite positivity rates and covariates.** Red font denotes zero positivity rate extrapolated from previous recording of parasitaemia or derived from published figures rather than text. Click here for file

Additional file 6 **Forest plots of Day 2 parasite positivity rates in Africa.** Estimates and 95% CI are shown by treatment, sorted by year in descending order (most recent first). Heterogeneity between studies I^2^ : AL = 92.3%; AS+AQ = 92.9%; AS+MQ = 91.7%; AS+SP = 95.6%; PIP+DHA = 60.9%. Click here for file

Additional file 7 **Forest plots of Day 2 parasite positivity rates in Asia.** Estimates and 95% CI are shown by treatment, sorted by year in descending order (most recent first). Heterogeneity between studies I^2^ : AL = 90.3%; AS+AQ = 93.8%; AS+MQ = 95.3%; AS+SP = 42.1%; PIP+DHA = 96.0%. Click here for file

Additional file 8 Studies with Day 3 parasitaemia positivity rates ≥10%. Click here for file
