# Supplementary material for: Early parasitological response following artemisinin-containing regimens: a critical review of the literature
Source: Malar J. 2013 Apr 19;12:125. doi: 10.1186/1475-2875-12-125 (PMC3649884; doi:10.1186/1475-2875-12-125)
Supplement: Additional file 1 — PubMed search terms. [file 1475-2875-12-125-S1.pdf]

PubMed search terms:

(malaria OR plasmodium)

AND

(amodiaquine OR atovaquone OR artemisinin OR arteether OR artesunate OR artemether OR artemether OR artemotil OR azithromycin OR artekin OR chloroquine OR chlorproguanil OR cycloguanil OR clindamycin OR coartem OR dapsone OR dihydroartemisinin OR duo-cotecxin OR doxycycline OR halofantrine OR lumefantrine OR lariam OR malarone OR mefloquine OR naphthoquine OR naphthoquinone OR piperaquine OR primaquine OR proguanil OR pyrimethamine OR pyronaridine OR proguanil OR quinidine OR quinine OR riamet OR sulphadoxine OR tetracycline OR tafenoquine)
