# Supplementary material for: Early parasitological response following artemisinin-containing regimens: a critical review of the literature
Source: Malar J. 2013 Apr 19;12:125. doi: 10.1186/1475-2875-12-125 (PMC3649884; doi:10.1186/1475-2875-12-125)
Supplement: Additional file 2 — Study references included in the analysis. [file 1475-2875-12-125-S2.pdf]

Additional File 2 : References

Authors	Year	Title	Journal	Volume	Issue	Pages	Accession No.
4ABC Study Group	2011	A head-to-head comparison of four artemisinin-based combinations for treating uncomplicated malaria in African children: a randomized trial	PLoS medicine	8	11	e1001119	22087077
Abacassamo et al.	2004	Efficacy of chloroquine, amodiaquine, sulphadoxine-pyrimethamine and combination therapy with artesunate in Mozambican children with non-complicated malaria	Tropical medicine & international health : TM & IH	9	2	200-8	15040556
Abdulla et al.	2008	Efficacy and safety of artemether-lumefantrine dispersible tablets compared with crushed commercial tablets in African infants and children with uncomplicated malaria: a randomised, single-blind, multicentre trial	Lancet	372	9652	1819-27	18926569
Achan et al.	2009	Effectiveness of quinine versus artemether-lumefantrine for treating uncomplicated falciparum malaria in Ugandan children: randomised trial	BMJ (Clinical research ed.)	339		b2763	19622553
Adam et al.	2006	A fixed-dose 24-hour regimen of artesunate plus sulfamethoxypyrazine-pyrimethamine for the treatment of uncomplicated Plasmodium falciparum malaria in eastern Sudan	Annals of clinical microbiology and antimicrobials	5		18	16934158
Adam et al.	2005a	Efficacies of mefloquine alone and of artesunate followed by mefloquine, for the treatment of uncomplicated, Plasmodium falciparum malaria in eastern Sudan	Annals of tropical medicine and parasitology	99	2	111-7	15814029
Adam et al.	2005b	A comparison of the efficacy of artesunate plus sulfadoxine-pyrimethamine with that of sulfadoxine-pyrimethamine alone, in the treatment of uncomplicated, Plasmodium falciparum malaria in eastern Sudan	Annals of tropical medicine and parasitology	99	5	449-55	16004704
Adjei et al.	2008	Amodiaquine-artesunate vs artemether-lumefantrine for uncomplicated malaria in Ghanaian children: a randomized efficacy and safety trial with one year follow-up	Malaria journal	7		127	18620577
Adjuik et al.	2002	Amodiaquine-artesunate versus amodiaquine for uncomplicated Plasmodium falciparum malaria in African children: a randomised, multicentre trial	Lancet	359	9315	1365-72	11978332
Agomo et al.	2008	Efficacy, safety and tolerability of artesunate-mefloquine in the treatment of uncomplicated Plasmodium falciparum malaria in four geographic zones of Nigeria	Malaria journal	7		172	18782445
Alecrim et al.	2006	Successful treatment of Plasmodium falciparum malaria with a six-dose regimen of artemether-lumefantrine versus quinine-doxycycline in the Western Amazon region of Brazil	The American journal of tropical medicine and hygiene	74	1	20-5	16407341
Allen et al.	2009	Efficacy of sulphadoxine-pyrimethamine with or without artesunate for the treatment of uncomplicated Plasmodium falciparum malaria in southern Mozambique: a randomized controlled trial	Malaria journal	8		141	19558654
Asante et al.	2009	Adherence to Artesunate-Amodiaquine Therapy for Uncomplicated Malaria in Rural Ghana: A Randomised Trial of Supervised versus Unsupervised Drug Administration	Journal of tropical medicine	2009		529583	20339565
Ashley et al.	2004	Randomized, controlled dose-optimization studies of dihydroartemisinin-piperaquine for the treatment of uncomplicated multidrug-resistant falciparum malaria in Thailand	The Journal of infectious diseases	190	10	1773-82	15499533
Ashley et al.	2005	A randomized, controlled study of a simple, once-daily regimen of dihydroartemisinin-piperaquine for the treatment of uncomplicated, multidrug-resistant falciparum malaria	Clinical infectious diseases : an official publication of the Infectious Diseases Society of America	41	4	425-32	16028147
Ashley et al.	2006	An open label randomized comparison of mefloquine-artesunate as separate tablets vs. a new co-formulated combination for the treatment of uncomplicated multidrug-resistant falciparum malaria in Thailand	Tropical medicine & international health : TM & IH	11	11	1653-60	17054744
Ashley et al.	2007	Pharmacokinetic study of artemether-lumefantrine given once daily for the treatment of uncomplicated multidrug-resistant falciparum malaria	Tropical medicine & international health : TM & IH	12	2	201-8	17300626
Asih et al.	2009	Efficacy of artemisinin-based combination therapy for treatment of persons with uncomplicated Plasmodium falciparum malaria in West Sumba District, East Nusa Tenggara Province, Indonesia, and genotypic profiles of the parasite	The American journal of tropical medicine and hygiene	80	6	914-8	19478248
Assefa et al.	2010	Therapeutic efficacy of Artemether/Lumefantrine (Coartem(R)) against Plasmodium falciparum in Kersa, South West Ethiopia	Parasites & vectors	3	1	1	20051120
Avila et al.	2004	Efficacy of mefloquine and mefloquine-artesunate for the treatment of uncomplicated Plasmodium falciparum malaria in the Amazon region of Bolivia	Tropical medicine & international health : TM & IH	9	2	217-21	15040558
Ayede et al.	2010	An open randomized clinical trial in comparing two artesunate-based combination treatments on Plasmodium falciparum malaria in Nigerian children: artesunate/sulphamethoxypyrazine/pyrimethamine (fixed dose over 24 hours) versus artesunate/amodiaquine (fixed dose over 48 hours)	Malaria journal	9		378	21194422

Additional File 2 : References

Authors	Year	Title	Journal	Volume	Issue	Pages	Accession No.
Barennes et al.	2004	A randomized trial of amodiaquine and artesunate alone and in combination for the treatment of uncomplicated falciparum malaria in children from Burkina Faso	Tropical medicine & international health : TM & IH	9	4	438-44	15078261
Bassat et al.	2009	Dihydroartemisinin-piperaquine and artemether-lumefantrine for treating uncomplicated malaria in African children: a randomised, non-inferiority trial	PloS one	4	11	e7871	19936217
Bell et al.	2008	Sulfadoxine-pyrimethamine-based combinations for malaria: a randomised blinded trial to compare efficacy, safety and selection of resistance in Malawi	PloS one	3	2	e1578	18270569
Bell et al.	2009	Measurement of adherence, drug concentrations and the effectiveness of artemether-lumefantrine, chlorproguanil-dapsone or sulphadoxine-pyrimethamine in the treatment of uncomplicated malaria in Malawi	Malaria journal	8		204	19709418
Bethell et al.	2011	Artesunate dose escalation for the treatment of uncomplicated malaria in a region of reported artemisinin resistance: a randomized clinical trial	PloS one	6	5	e19283	21603629
Blair et al.	2006	Therapeutic efficacy test in malaria falciparum in Antioquia, Colombia	Malaria journal	5		14	16504002
Bonnet et al.	2007	Efficacy of antimalarial treatment in Guinea: in vivo study of two artemisinin combination therapies in Dabola and molecular markers of resistance to sulphadoxine-pyrimethamine in N'Zerekore	Malaria journal	6		54	17477865
Bonnet et al.	2009	Varying efficacy of artesunate+amodiaquine and artesunate+sulphadoxine-pyrimethamine for the treatment of uncomplicated falciparum malaria in the Democratic Republic of Congo: a report of two in-vivo studies	Malaria journal	8		192	19664280
Borrmann et al.	2005	Short-course regimens of artesunate-fosmidomycin in treatment of uncomplicated Plasmodium falciparum malaria	Antimicrobial agents and chemotherapy	49	9	3749-54	16127049
Borrmann et al.	2011	Declining responsiveness of Plasmodium falciparum infections to artemisinin-based combination treatments on the Kenyan coast	PloS one	6	11	e26005	22102856
Bousema et al.	2006	Moderate effect of artemisinin-based combination therapy on transmission of Plasmodium falciparum	The Journal of infectious diseases	193	8	1151-9	16544256
Bouyou-Akotet et al.	2010	Efficacy and safety of a new pediatric artesunate-mefloquine drug formulation for the treatment of uncomplicated falciparum malaria in Gabon	Wiener klinische Wochenschrift	122	5-6	173-8	20361381
Bukirwa et al.	2006	Artemisinin combination therapies for treatment of uncomplicated malaria in Uganda	PLoS clinical trials	1	1	e7	16871329
Campbell et al.	2006	A randomized trial comparing the efficacy of four treatment regimens for uncomplicated falciparum malaria in Assam state, India	Transactions of the Royal Society of Tropical Medicine and Hygiene	100	2	108-18	16289651
Chanda et al.	2006	Assessment of the therapeutic efficacy of a paediatric formulation of artemether-lumefantrine (Coartesiane) for the treatment of uncomplicated Plasmodium falciparum in children in Zambia	Malaria journal	5		75	16938133
Charle et al.	2009	Efficacy of Artesunate + Sulphadoxine-Pyrimethamine (AS + SP) and Amodiaquine + Sulphadoxine-Pyrimethamine (AQ + SP) for Uncomplicated falciparum Malaria in Equatorial Guinea (Central Africa)	Journal of tropical medicine	2009		781865	20339460
de Oliveira et al.	2011	Efficacy and effectiveness of mefloquine and artesunate combination therapy for uncomplicated Plasmodium falciparum malaria in the Peruvian Amazon	The American journal of tropical medicine and hygiene	85	3	573-8	21896825
de Vries et al.	2000	Combinations of artemisinin and quinine for uncomplicated falciparum malaria: efficacy and pharmacodynamics	Antimicrobial agents and chemotherapy	44	5	1302-8	10770766
Denis et al.	2002	Efficacy and safety of dihydroartemisinin-piperaquine (Artekin) in Cambodian children and adults with uncomplicated falciparum malaria	Clinical infectious diseases : an official publication of the Infectious Diseases Society of America	35	12	1469-76	12471565
Denis et al.	2006a	Surveillance of the efficacy of artesunate and mefloquine combination for the treatment of uncomplicated falciparum malaria in Cambodia	Tropical medicine & international health : TM & IH	11	9	1360-6	16930257
Denis et al.	2006b	Efficacy of artemether-lumefantrine for the treatment of uncomplicated falciparum malaria in northwest Cambodia	Tropical medicine & international health : TM & IH	11	12	1800-7	17176344
Depoortere et al.	2005	Efficacy and effectiveness of the combination of sulfadoxine/pyrimethamine and a 3-day course of artesunate for the treatment of uncomplicated falciparum malaria in a refugee settlement in Zambia	Tropical medicine & international health : TM & IH	10	2	139-45	15679556
Diem Thuy et al.	2007	Clinical efficacy of high dose monotherapy of oral dihydroartemisinin in uncomplicated falciparum malaria in viet nam	Japanese journal of infectious diseases	60	4	161-6	17642523

Additional File 2 : References

Authors	Year	Title	Journal	Volume	Issue	Pages	Accession No.
Djimdé et al.	2008	Efficacy, safety, and selection of molecular markers of drug resistance by two ACTs in Mali	The American journal of tropical medicine and hygiene	78	3	455-61	18337343
Dondorp et al.	2009	Artemisinin resistance in Plasmodium falciparum malaria	The New England journal of medicine	361	5	455-67	19641202
Dunyo et al.	2011	Randomized trial of safety and effectiveness of chlorproguanil-dapsone and lumefantrine-artemether for uncomplicated malaria in children in the Gambia	PloS one	6	6	e17371	21666744
Durrani et al.	2005	Efficacy of combination therapy with artesunate plus amodiaquine compared to monotherapy with chloroquine, amodiaquine or sulfadoxine-pyrimethamine for treatment of uncomplicated Plasmodium falciparum in Afghanistan	Tropical medicine & international health : TM & IH	10	6	521-9	15941414
Elamin et al.	2010	Descriptive study on the efficacy of artemether-lumefantrine in the treatment of uncomplicated Plasmodium falciparum malaria in Sudan	European journal of clinical pharmacology	66	3	231-7	20187287
Falade et al.	2005	Efficacy and safety of artemether-lumefantrine (Coartem) tablets (six-dose regimen) in African infants and children with acute, uncomplicated falciparum malaria	Transactions of the Royal Society of Tropical Medicine and Hygiene	99	6	459-67	15837358
Falade et al.	2008a	High efficacy of two artemisinin-based combinations (artemether-lumefantrine and artesunate plus amodiaquine) for acute uncomplicated malaria in Ibadan, Nigeria	Tropical medicine & international health : TM & IH	13	5	635-43	18346028
Falade et al.	2008b	Evaluation of the efficacy and safety of artemether-lumefantrine in the treatment of acute uncomplicated Plasmodium falciparum malaria in Nigerian infants and children	Malaria journal	7		246	19038036
Fanello et al.	2007	A randomised trial to assess the safety and efficacy of artemether-lumefantrine (Coartem) for the treatment of uncomplicated Plasmodium falciparum malaria in Rwanda	Transactions of the Royal Society of Tropical Medicine and Hygiene	101	4	344-50	17005222
Fanello et al.	2008	A randomised trial to assess the efficacy and safety of chlorproguanil/dapsone + artesunate for the treatment of uncomplicated Plasmodium falciparum malaria	Transactions of the Royal Society of Tropical Medicine and Hygiene	102	5	412-20	18328518
Faucher et al.	2009	Comparison of sulfadoxine-pyrimethamine, unsupervised artemether-lumefantrine, and unsupervised artesunate-amodiaquine fixed-dose formulation for uncomplicated plasmodium falciparum malaria in Benin: a randomized effectiveness noninferiority trial	The Journal of infectious diseases	200	1	57-65	19469703
Faye et al.	2007	Efficacy and tolerability of four antimalarial combinations in the treatment of uncomplicated Plasmodium falciparum malaria in Senegal	Malaria journal	6		80	17570848
Faye et al.	2010a	A randomized trial of artesunate mefloquine versus artemether lumefantrine for the treatment of uncomplicated Plasmodium falciparum malaria in Senegalese children	The American journal of tropical medicine and hygiene	82	1	140-4	20065010
Faye et al.	2010b	Efficacy and tolerability of artesunate-amodiaquine (Camoquin plus) versus artemether-lumefantrine (Coartem) against uncomplicated Plasmodium falciparum malaria: multisite trial in Senegal and Ivory Coast	Tropical medicine & international health : TM & IH	15	5	608-13	20214761
Fehintola et al.	2008	Effects of artesunate-cotrimoxazole and amodiaquine-artesunate against asexual and sexual stages of Plasmodium falciparum malaria in Nigerian children	Journal of infection and chemotherapy : official journal of the Japan Society of Chemotherapy	14	3	188-94	18574653
Fehintola et al.	2010	Comparative study of efficacy of artesunate plus cotrimoxazole and artesunate plus chloroquine in the treatment of malaria in Nigerian children: a preliminary report	Journal of vector borne diseases	47	3	145-50	20834083
Gbotosho et al.	2011a	Therapeutic efficacy and effects of artemether-lumefantrine and artesunate-amodiaquine coformulated or copackaged on malaria-associated anemia in children with uncomplicated Plasmodium falciparum malaria in Southwest Nigeria	The American journal of tropical medicine and hygiene	84	5	813-9	21540395
Gbotosho et al.	2011b	A Simple Dose Regimen of Artesunate and Amodiaquine Based on Age or Body Weight Range for Uncomplicated Falciparum Malaria in Children: Comparison of Therapeutic Efficacy With Standard Dose Regimen of Artesunate and Amodiaquine and Artemether-Lumefantrine	American journal of therapeutics				21519221
Giao et al.	2001	Artemisinin for treatment of uncomplicated falciparum malaria: is there a place for monotherapy?	The American journal of tropical medicine and hygiene	65	6	690-5	11791958
Giao et al.	2004	CV8, a new combination of dihydroartemisinin, piperaquine, trimethoprim and primaquine, compared with atovaquone-proguanil against falciparum malaria in Vietnam	Tropical medicine & international health : TM & IH	9	2	209-16	15040557
Gil et al.	2003	Efficacy of artesunate plus chloroquine for uncomplicated malaria in children in Sao Tome and Principe: a double-blind, randomized, controlled trial	Transactions of the Royal Society of Tropical Medicine and Hygiene	97	6	703-6	16117967
Gomez et al.	2003	Randomised efficacy and safety study of two 3-day artesunate rectal capsule/mefloquine regimens versus artesunate alone for uncomplicated malaria in Ecuadorian children	Acta tropica	89	1	47-53	14636982
Grande et al.	2007	A randomised controlled trial to assess the efficacy of dihydroartemisinin-piperaquine for the treatment of uncomplicated falciparum malaria in Peru	PloS one	2	10	e1101	17971864

Additional File 2 : References

Authors	Year	Title	Journal	Volume	Issue	Pages	Accession No.
Grandesso et al.	2006	Low efficacy of the combination artesunate plus amodiaquine for uncomplicated falciparum malaria among children under 5 years in Kailahun, Sierra Leone	Tropical medicine & international health : TM & IH	11	7	1017-21	16827702
Guthmann et al.	2005	Antimalarial efficacy of chloroquine, amodiaquine, sulfadoxine-pyrimethamine, and the combinations of amodiaquine + artesunate and sulfadoxine-pyrimethamine + artesunate in Huambo and Bie provinces, central Angola	Transactions of the Royal Society of Tropical Medicine and Hygiene	99	7	485-92	15876443
Gutman et al.	2009	Mefloquine pharmacokinetics and mefloquine-artesunate effectiveness in Peruvian patients with uncomplicated Plasmodium falciparum malaria	Malaria journal	8		58	19358697
Hamour et al.	2005	Malaria in the Nuba Mountains of Sudan: baseline genotypic resistance and efficacy of the artesunate plus sulfadoxine-pyrimethamine and artesunate plus amodiaquine combinations	Transactions of the Royal Society of Tropical Medicine and Hygiene	99	7	548-54	15869770
Haque et al.	2007	Therapeutic efficacy of artemether-lumefantrine for the treatment of uncomplicated Plasmodium falciparum malaria in Bangladesh	The American journal of tropical medicine and hygiene	76	1	39-41	17255226
Hasugian et al.	2007	Dihydroartemisinin-Piperaquine versus Artesunate-Amodiaquine: Superior Efficacy and Posttreatment Prophylaxis against Multidrug-Resistant *Plasmodium falciparum* and *Plasmodium vivax* Malaria	Clinical infectious diseases : an official publication of the Infectious Diseases Society of America	44		1067-74	17366451
Hatz et al.	2008	Treatment of acute uncomplicated falciparum malaria with artemether-lumefantrine in nonimmune populations: a safety, efficacy, and pharmacokinetic study	The American journal of tropical medicine and hygiene	78	2	241-7	18256423
Hien et al.	2004	Dihydroartemisinin-piperaquine against multidrug-resistant Plasmodium falciparum malaria in Vietnam: randomised clinical trial	Lancet	363	9402	18-22	14723988
Hombhanje et al.	2009	Artemisinin-naphthoquine combination (ARCO) therapy for uncomplicated falciparum malaria in adults of Papua New Guinea: a preliminary report on safety and efficacy	Malaria journal	8		196	19671190
Hung et al.	2004	Artesunate with mefloquine at various intervals for non-severe Plasmodium falciparum malaria	The American journal of tropical medicine and hygiene	71	2	160-6	15306704
Huong et al.	2001	Resistance of Plasmodium falciparum to antimalarial drugs in a highly endemic area of southern Viet Nam: a study in vivo and in vitro	Transactions of the Royal Society of Tropical Medicine and Hygiene	95	3	325-9	11491008
Hutagalung et al.	2005	A randomized trial of artemether-lumefantrine versus mefloquine-artesunate for the treatment of uncomplicated multi-drug resistant Plasmodium falciparum on the western border of Thailand	Malaria journal	4		46	16179089
Hwang et al.	2011	In vivo efficacy of artemether-lumefantrine against uncomplicated Plasmodium falciparum malaria in Central Ethiopia	Malaria journal	10		209	21798054
Ibrahium et al.	2007	Efficacies of artesunate plus either sulfadoxine-pyrimethamine or amodiaquine, for the treatment of uncomplicated, Plasmodium falciparum malaria in eastern Sudan	Annals of tropical medicine and parasitology	101	1	15-21	17244406
Janssens et al.	2007	A randomized open study to assess the efficacy and tolerability of dihydroartemisinin-piperaquine for the treatment of uncomplicated falciparum malaria in Cambodia	Tropical medicine & international health : TM & IH	12	2	251-9	17300633
Juma et al.	2008	A randomized, open-label, comparative efficacy trial of artemether-lumefantrine suspension versus artemether-lumefantrine tablets for treatment of uncomplicated Plasmodium falciparum malaria in children in western Kenya	Malaria journal	7		262	19102746
Kabanywanyi et al.	2007	Efficacy and safety of artemisinin-based antimalarial in the treatment of uncomplicated malaria in children in southern Tanzania	Malaria journal	6		146	17996121
Kamya et al.	2007	Artemether-lumefantrine versus dihydroartemisinin-piperaquine for treatment of malaria: a randomized trial	PLoS clinical trials	2	5	e20	17525792
Karema et al.	2006	Safety and efficacy of dihydroartemisinin/piperaquine (Artekin) for the treatment of uncomplicated Plasmodium falciparum malaria in Rwandan children	Transactions of the Royal Society of Tropical Medicine and Hygiene	100	12	1105-11	16766006
Karunajeewa et al.	2003	Safety and therapeutic efficacy of artesunate suppositories for treatment of malaria in children in Papua New Guinea	The Pediatric infectious disease journal	22	3	251-6	12634587
Karunajeewa et al.	2008a	Pharmacokinetics and efficacy of piperaquine and chloroquine in Melanesian children with uncomplicated malaria	Antimicrobial agents and chemotherapy	52	1	237-43	17967917
Karunajeewa et al.	2008b	A trial of combination antimalarial therapies in children from Papua New Guinea	The New England journal of medicine	359	24	2545-57	19064624
Kayentao et al.	2009	Artemisinin-based combinations versus amodiaquine plus sulphadoxine-pyrimethamine for the treatment of uncomplicated malaria in Faladje, Mali	Malaria journal	8		5	19128455

Additional File 2 : References

Authors	Year	Title	Journal	Volume	Issue	Pages	Accession No.
Kobbe et al.	2008	A randomized trial on effectiveness of artemether-lumefantrine versus artesunate plus amodiaquine for unsupervised treatment of uncomplicated Plasmodium falciparum malaria in Ghanaian children	Malaria journal	7		261	19099594
Kofoed et al.	2003	No benefits from combining chloroquine with artesunate for three days for treatment of Plasmodium falciparum in Guinea-Bissau	Transactions of the Royal Society of Tropical Medicine and Hygiene	97	4	429-33	15259473
Koram et al.	2005	Comparative efficacy of antimalarial drugs including ACTs in the treatment of uncomplicated malaria among children under 5 years in Ghana	Acta tropica	95	3	194-203	16054584
Koram et al.	2008	Efficacy of amodiaquine/artesunate combination therapy for uncomplicated malaria in children under five years in ghana	Ghana medical journal	42	2	55-60	19180204
Krudsood et al.	2000	A randomized clinical trial of combinations of artesunate and azithromycin for treatment of uncomplicated Plasmodium falciparum malaria in Thailand	The Southeast Asian journal of tropical medicine and public health	31	4	801-7	11414432
Krudsood et al.	2002	Artesunate and mefloquine given simultaneously for three days via a prepacked blister is equally effective and tolerated as a standard sequential treatment of uncomplicated acute Plasmodium falciparum malaria: randomized, double-blind study in Thailand	The American journal of tropical medicine and hygiene	67	5	465-72	12479545
Krudsood et al.	2003	Comparative clinical trial of two-fixed combinations dihydroartemisinin-napthoquine-trimethoprim (DNP) and artemether-lumefantrine (Coartem/Riamet) in the treatment of acute uncomplicated falciparum malaria in Thailand	The Southeast Asian journal of tropical medicine and public health	34	2	316-21	12971556
Krudsood et al.	2007	Dose ranging studies of new artemisinin-piperaquine fixed combinations compared to standard regimens of artemisisnin combination therapies for acute uncomplicated falciparum malaria	The Southeast Asian journal of tropical medicine and public health	38	6	971-8	18613536
Kshirsagar et al.	2000	A randomized, double-blind, parallel-group, comparative safety, and efficacy trial of oral co-artemether versus oral chloroquine in the treatment of acute uncomplicated Plasmodium falciparum malaria in adults in India	The American journal of tropical medicine and hygiene	62	3	402-8	11037786
Lefevre et al.	2001	A clinical and pharmacokinetic trial of six doses of artemether-lumefantrine for multidrug-resistant Plasmodium falciparum malaria in Thailand	The American journal of tropical medicine and hygiene	64	5-6	247-56	11463111
Marquino et al.	2003	Efficacy of mefloquine and a mefloquine-artesunate combination therapy for the treatment of uncomplicated Plasmodium falciparum malaria in the Amazon Basin of Peru	The American journal of tropical medicine and hygiene	68	5	608-12	12812355
Marquino et al.	2005	Efficacy and tolerability of artesunate plus sulfadoxine-pyrimethamine and sulfadoxine-pyrimethamine alone for the treatment of uncomplicated Plasmodium falciparum malaria in Peru	The American journal of tropical medicine and hygiene	72	5	568-72	15891131
Martensson et al.	2005	Efficacy of artesunate plus amodiaquine versus that of artemether-lumefantrine for the treatment of uncomplicated childhood Plasmodium falciparum malaria in Zanzibar, Tanzania	Clinical infectious diseases : an official publication of the Infectious Diseases Society of America	41	8	1079-86	16163624
Massougbodji et al.	2002	A randomized, double-blind study on the efficacy and safety of a practical three-day regimen with artesunate and mefloquine for the treatment of uncomplicated Plasmodium falciparum malaria in Africa	Transactions of the Royal Society of Tropical Medicine and Hygiene	96	6	655-9	12625145
Mayxay et al.	2004	Randomized comparison of chloroquine plus sulfadoxine-pyrimethamine versus artesunate plus mefloquine versus artemether-lumefantrine in the treatment of uncomplicated falciparum malaria in the Lao People's Democratic Republic	Clinical infectious diseases : an official publication of the Infectious Diseases Society of America	39	8	1139-47	15486837
Mayxay et al.	2006	An open, randomized comparison of artesunate plus mefloquine vs. dihydroartemisinin-piperaquine for the treatment of uncomplicated Plasmodium falciparum malaria in the Lao People's Democratic Republic (Laos)	Tropical medicine & international health : TM & IH	11	8	1157-65	16903879
Menan et al.	2011	Comparative study of the efficacy and tolerability of dihydroartemisinin-piperaquine-trimethoprim versus artemether-lumefantrine in the treatment of uncomplicated Plasmodium falciparum malaria in Cameroon, Ivory Coast and Senegal	Malaria journal	10		185	21740570
Ménard et al.	2007	Randomized clinical trial of artemisinin versus non-artemisinin combination therapy for uncomplicated falciparum malaria in Madagascar	Malaria journal	6		65	17519010
Ménard et al.	2008	Assessment of the efficacy of antimalarial drugs recommended by the National Malaria Control Programme in Madagascar: up-dated baseline data from randomized and multi-site clinical trials	Malaria journal	7		55	18394169

Additional File 2 : References

Authors	Year	Title	Journal	Volume	Issue	Pages	Accession No.
Mens et al.	2008	A randomized trial to monitor the efficacy and effectiveness by QT-NASBA of artemether-lumefantrine versus dihydroartemisinin-piperaquine for treatment and transmission control of uncomplicated Plasmodium falciparum malaria in western Kenya	Malaria journal	7		237	19017387
Meremikwu et al.	2006	Artemether-lumefantrine versus artesunate plus amodiaquine for treating uncomplicated childhood malaria in Nigeria: randomized controlled trial	Malaria journal	5		43	16704735
Michael et al.	2010	Early variations in plasmodium falciparum dynamics in Nigerian children after treatment with two artemisinin-based combinations: implications on delayed parasite clearance	Malaria journal	9		335	21092220
Mockenhaupt et al.	2005	A randomized, placebo-controlled, double-blind trial on sulfadoxine-pyrimethamine alone or combined with artesunate or amodiaquine in uncomplicated malaria	Tropical medicine & international health : TM & IH	10	6	512-20	15941413
Mohamed et al.	2006	The efficacies of artesunate-sulfadoxine-pyrimethamine and artemether-lumefantrine in the treatment of uncomplicated, Plasmodium falciparum malaria, in an area of low transmission in central Sudan	Annals of tropical medicine and parasitology	100	1	5-10	16417707
Mukhtar et al.	2007	A comparative study on the efficacy of artesunate plus sulphadoxine/pyrimethamine versus artemether-lumefantrine in eastern Sudan	Malaria journal	6		92	17631681
Mulenga et al.	2006	Safety and efficacy of lumefantrine-artemether (Coartem) for the treatment of uncomplicated Plasmodium falciparum malaria in Zambian adults	Malaria journal	5		73	16923176
Mutabingwa et al.	2005	Amodiaquine alone, amodiaquine+sulfadoxine-pyrimethamine, amodiaquine+artesunate, and artemether-lumefantrine for outpatient treatment of malaria in Tanzanian children: a four-arm randomised effectiveness trial	Lancet	365	9469	1474-80	15850631
Na-Bangchang et al.	2010	Declining in efficacy of a three-day combination regimen of mefloquine-artesunate in a multi-drug resistance area along the Thai-Myanmar border	Malaria journal	9		273	20929590
Nahum et al.	2007	Adding artesunate to sulphadoxine-pyrimethamine greatly improves the treatment efficacy in children with uncomplicated falciparum malaria on the coast of Benin, West Africa	Malaria journal	6		170	18154655
Nambei et al.	2005	Efficacy comparison between anti-malarial drugs in Africans presenting with mild malaria in the Central Republic of Africa: a preliminary study	Parasite (Paris France)	12	1	73-7	15828586
Nambozi et al.	2011	Safety and efficacy of dihydroartemisinin-piperaquine versus artemether-lumefantrine in the treatment of uncomplicated Plasmodium falciparum malaria in Zambian children	Malaria journal	10		50	21352609
Ndayiragije et al.	2004	Efficacy of therapeutic combinations with artemisinin derivatives in the treatment of non complicated malaria in Burundi	Tropical medicine & international health : TM & IH	9	6	673-9	15189457
Ndiaye et al.	2008	Randomized, comparative study of the efficacy and safety of artesunate plus amodiaquine, administered as a single daily intake versus two daily intakes in the treatment of uncomplicated falciparum malaria	Malaria journal	7		16	18205945
Ndiaye et al.	2009	Randomized, multicentre assessment of the efficacy and safety of ASAQ--a fixed-dose artesunate-amodiaquine combination therapy in the treatment of uncomplicated Plasmodium falciparum malaria	Malaria journal	8		125	19505304
Ngasala et al.	2011a	Effectiveness of artemether-lumefantrine provided by community health workers in under-five children with uncomplicated malaria in rural Tanzania: an open label prospective study	Malaria journal	10		64	21410954
Ngasala et al.	2011b	Efficacy and effectiveness of artemether-lumefantrine after initial and repeated treatment in children <5 years of age with acute uncomplicated Plasmodium falciparum malaria in rural Tanzania: a randomized trial	Clinical infectious diseases : an official publication of the Infectious Diseases Society of America	52	7	873-82	21427394
Nguyen et al.	2003	Treatment of uncomplicated falciparum malaria in southern Vietnam: can chloroquine or sulfadoxine-pyrimethamine be reintroduced in combination with artesunate?	Clinical infectious diseases : an official publication of the Infectious Diseases Society of America	37	11	1461-6	14614668
Noedl et al.	2006	Azithromycin combination therapy with artesunate or quinine for the treatment of uncomplicated Plasmodium falciparum malaria in adults: a randomized, phase 2 clinical trial in Thailand	Clinical infectious diseases : an official publication of the Infectious Diseases Society of America	43	10	1264-71	17051490
Noedl et al.	2010	Artemisinin resistance in Cambodia: a clinical trial designed to address an emerging problem in Southeast Asia	Clinical infectious diseases : an official publication of the Infectious Diseases Society of America	51	11	e82-9	21028985
Obonyo et al.	2003	Artesunate plus sulfadoxine-pyrimethamine for uncomplicated malaria in Kenyan children: a randomized, double-blind, placebo-controlled trial	Transactions of the Royal Society of Tropical Medicine and Hygiene	97	5	585-91	15307433
Oduro et al.	2004	A randomized, comparative study of two regimens of beta-artemether for the treatment of uncomplicated, Plasmodium falciparum malaria, in northern Ghana	Annals of tropical medicine and parasitology	98	5	433-40	15257791

Additional File 2 : References

Authors	Year	Title	Journal	Volume	Issue	Pages	Accession No.
Oduro et al.	2008	A randomized, comparative study of supervised and unsupervised artesunate-amodiaquine, for the treatment of uncomplicated malaria in Ghana	Annals of tropical medicine and parasitology	102	7	565-76	18817597
Osorio et al.	2007	Artemisinin-based combination therapy for uncomplicated Plasmodium falciparum malaria in Colombia	Malaria journal	6		25	17328806
Owusu-Agyei et al.	2008	An open label, randomised trial of artesunate+amodiaquine, artesunate+chlorproguanil-dapsone and artemether-lumefantrine for the treatment of uncomplicated malaria	PloS one	3	6	e2530	18575626
Oyakhirome et al.	2007	Artesunate--amodiaquine combination therapy for falciparum malaria in young Gabonese children	Malaria journal	6		29	17352806
Penali et al.	2008	Single-day, three-dose treatment with fixed dose combination artesunate/sulfamethoxypyrazine/pyrimethamine to cure Plasmodium falciparum malaria	International journal of infectious diseases : IJID : official publication of the International Society for Infectious Diseases	12	4	430-7	18343702
Piola et al.	2005	Supervised versus unsupervised intake of six-dose artemether-lumefantrine for treatment of acute, uncomplicated Plasmodium falciparum malaria in Mbarara, Uganda: a randomised trial	Lancet	365	9469	1467-73	15850630
Premji et al.	2009	Chlorproguanil-dapsone-artesunate versus artemether-lumefantrine: a randomized, double-blind phase III trial in African children and adolescents with uncomplicated Plasmodium falciparum malaria	PloS one	4	8	e6682	19690618
Priotto et al.	2003	Artesunate and sulfadoxine-pyrimethamine combinations for the treatment of uncomplicated Plasmodium falciparum malaria in Uganda: a randomized, double-blind, placebo-controlled trial	Transactions of the Royal Society of Tropical Medicine and Hygiene	97	3	325-30	15228253
Pukrittayakamee et al.	2004	Activities of artesunate and primaquine against asexual- and sexual-stage parasites in falciparum malaria	Antimicrobial agents and chemotherapy	48	4	1329-34	15047537
Rahman et al.	2008	Adherence and efficacy of supervised versus non-supervised treatment with artemether/lumefantrine for the treatment of uncomplicated Plasmodium falciparum malaria in Bangladesh: a randomised controlled trial	Transactions of the Royal Society of Tropical Medicine and Hygiene	102	9	861-7	18606428
Ramharter et al.	2005	Artesunate-clindamycin versus quinine-clindamycin in the treatment of Plasmodium falciparum malaria: a randomized controlled trial	Clinical infectious diseases : an official publication of the Infectious Diseases Society of America	40	12	1777-84	15909266
Rasheed et al.	2011	Efficacy and safety of artemehter-lumefantrine in uncomplicated falciparum malaria in Liberia	JPMA. The Journal of the Pakistan Medical Association	61	2	131-4	21375159
Ratcliff et al.	2007	Two fixed-dose artemisinin combinations for drug-resistant falciparum and vivax malaria in Papua, Indonesia: an open-label randomised comparison	Lancet	369	9563	757-65	17336652
Rojanawatsirivej et al.	2003	Monitoring the therapeutic efficacy of antimalarials against uncomplicated falciparum malaria in Thailand	The Southeast Asian journal of tropical medicine and public health	34	3	536-41	15115123
Rulisa et al.	2007	Comparison of different artemisinin-based combinations for the treatment of Plasmodium falciparum malaria in children in Kigali, Rwanda, an area of resistance to sulfadoxine-pyrimethamine: artesunate plus sulfadoxine/pyrimethamine versus artesunate plus sulfamethoxypyrazine/pyrimethamine	The American journal of tropical medicine and hygiene	77	4	612-6	17978058
Rwagacondo et al.	2003	Efficacy of amodiaquine alone and combined with sulfadoxine-pyrimethamine and of sulfadoxine pyrimethamine combined with artesunate	The American journal of tropical medicine and hygiene	68	6	743-7	12887037
Rwagacondo et al.	2004	Is amodiaquine failing in Rwanda? Efficacy of amodiaquine alone and combined with artesunate in children with uncomplicated malaria	Tropical medicine & international health : TM & IH	9	10	1091-8	15482401
Sagara et al.	2006	A randomized trial of artesunate-sulfamethoxypyrazine-pyrimethamine versus artemether-lumefantrine for the treatment of uncomplicated Plasmodium falciparum malaria in Mali	The American journal of tropical medicine and hygiene	75	4	630-6	17038684
Sagara et al.	2008	A randomized trial of artesunate-mefloquine versus artemether-lumefantrine for treatment of uncomplicated Plasmodium falciparum malaria in Mali	The American journal of tropical medicine and hygiene	79	5	655-61	18981499
Sagara et al.	2009	Efficacy and safety of a fixed dose artesunate-sulphamethoxypyrazine-pyrimethamine compared to artemether-lumefantrine for the treatment of uncomplicated falciparum malaria across Africa: a randomized multi-centre trial	Malaria journal	8		63	19366448
Schwarz et al.	2005	5-day nonobserved artesunate monotherapy for treating uncomplicated Falciparum malaria in young Gabonese children	The American journal of tropical medicine and hygiene	73	4	705-9	16222013

Additional File 2 : References

Authors	Year	Title	Journal	Volume	Issue	Pages	Accession No.
Silachamroon et al.	2005	An open, randomized trial of three-day treatment with artesunate combined with a standard dose of mefloquine divided over either two or three days, for acute, uncomplicated falciparum malaria	The Southeast Asian journal of tropical medicine and public health	36	3	591-6	16124422
Sirima et al.	2003	Efficacy of artesunate plus chloroquine for the treatment of uncomplicated malaria in children in Burkina Faso: a double-blind, randomized, controlled trial	Transactions of the Royal Society of Tropical Medicine and Hygiene	97	3	345-9	15228257
Sirima et al.	2009	The efficacy and safety of a new fixed-dose combination of amodiaquine and artesunate in young African children with acute uncomplicated Plasmodium falciparum	Malaria journal	8		48	19291301
Sirivichayakul et al.	2007	Comparative study of the effectiveness and pharmacokinetics of two rectal artesunate/oral mefloquine combination regimens for the treatment of uncomplicated childhood falciparum malaria	Annals of tropical paediatrics	27	1	17-24	17469728
Smithuis et al.	2006	Efficacy and effectiveness of dihydroartemisinin-piperaquine versus artesunate-mefloquine in falciparum malaria: an open-label randomised comparison	Lancet	367	9528	2075-85	16798391
Smithuis et al.	2010	Effectiveness of five artemisinin combination regimens with or without primaquine in uncomplicated falciparum malaria: an open-label randomised trial	The Lancet infectious diseases	10	10	673-81	20832366
Smithuis et al.	2004a	Optimising operational use of artesunate-mefloquine: a randomised comparison of four treatment regimens	Transactions of the Royal Society of Tropical Medicine and Hygiene	98	3	182-92	15024929
Smithuis et al.	2004b	Comparison of chloroquine, sulfadoxine/pyrimethamine, mefloquine and mefloquine-artesunate for the treatment of falciparum malaria in Kachin State, North Myanmar	Tropical medicine & international health : TM & IH	9	11	1184-90	15548314
Song et al.	2011	Randomized trials of artemisinin-piperaquine, dihydroartemisinin-piperaquine phosphate and artemether-lumefantrine for the treatment of multi-drug resistant falciparum malaria in Cambodia-Thailand border area	Malaria journal	10		231	21827706
Sowunmi et al.	2005	Open randomized study of artesunate-amodiaquine vs. chloroquine-pyrimethamine-sulfadoxine for the treatment of uncomplicated Plasmodium falciparum malaria in Nigerian children	Tropical medicine & international health : TM & IH	10	11	1161-70	16262741
Sowunmi et al.	2009	Therapeutic efficacy and effects of artesunate-mefloquine and mefloquine alone on malaria-associated anemia in children with uncomplicated Plasmodium falciparum malaria in southwest Nigeria	The American journal of tropical medicine and hygiene	81	6	979-86	19996425
Sowunmi et al.	2011	Therapeutic Efficacy of Artesunate-Amodiaquine Combinations and the Plasma and Saliva Concentrations of Desethylamodiaquine in Children With Acute Uncomplicated Plasmodium falciparum Malaria	American journal of therapeutics				21192244
Sowunmi et al.	2007a	Activities of amodiaquine, artesunate, and artesunate-amodiaquine against asexual- and sexual-stage parasites in falciparum malaria in children	Antimicrobial agents and chemotherapy	51	5	1694-9	17325222
Sowunmi et al.	2007b	Therapeutic efficacy and effects of artemether-lumefantrine and amodiaquine-sulfalene-pyrimethamine on gametocyte carriage in children with uncomplicated Plasmodium falciparum malaria in southwestern Nigeria	The American journal of tropical medicine and hygiene	77	2	235-41	17690392
Staedke et al.	2004	Combination treatments for uncomplicated falciparum malaria in Kampala, Uganda: randomised clinical trial	Lancet	364	9449	1950-7	15567011
Stohrer et al.	2004	Therapeutic efficacy of artemether-lumefantrine and artesunate-mefloquine for treatment of uncomplicated Plasmodium falciparum malaria in Luang Namtha Province, Lao People's Democratic Republic	Tropical medicine & international health : TM & IH	9	11	1175-83	15548313
Suputtamongkol et al.	2003	The efficacy of combined mefloquine-artesunate versus mefloquine-primaquine on subsequent development of Plasmodium falciparum gametocytemia	The American journal of tropical medicine and hygiene	68	5	620-3	12812357
Sutherland et al.	2003	The addition of artesunate to chloroquine for treatment of Plasmodium falciparum malaria in Gambian children delays, but does not prevent treatment failure	The American journal of tropical medicine and hygiene	69	1	19-25	12932091
Sutherland et al.	2005	Reduction of malaria transmission to Anopheles mosquitoes with a six-dose regimen of co-artemether	PLoS medicine	2	4	e92	15839740
Swarthout et al.	2006	Artesunate + amodiaquine and artesunate + sulphadoxine-pyrimethamine for treatment of uncomplicated malaria in Democratic Republic of Congo: a clinical trial with determination of sulphadoxine and pyrimethamine-resistant haplotypes	Tropical medicine & international health : TM & IH	11	10	1503-11	17002724
Sykes et al.	2009	Azithromycin plus artesunate versus artemether-lumefantrine for treatment of uncomplicated malaria in Tanzanian children: a randomized, controlled trial	Clinical infectious diseases : an official publication of the Infectious Diseases Society of America	49	8	1195-201	19769536

Additional File 2 : References

Authors	Year	Title	Journal	Volume	Issue	Pages	Accession No.
Tall et al.	2007	Efficacy of artesunate plus amodiaquine, artesunate plus sulfadoxine-pyrimethamine, and chloroquine plus sulfadoxine-pyrimethamine in patients with uncomplicated Plasmodium falciparum in the Comoros Union	Acta tropica	102	3	176-81	17568549
Tangpukdee et al.	2005	An open randomized clinical trial of Artekin vs artesunate-mefloquine in the treatment of acute uncomplicated falciparum malaria	The Southeast Asian journal of tropical medicine and public health	36	5	1085-91	16438129
Tangpukdee et al.	2008	Efficacy of Artequick versus artesunate-mefloquine in the treatment of acute uncomplicated falciparum malaria in Thailand	The Southeast Asian journal of tropical medicine and public health	39	1	1-8	18567436
Thanh et al.	2009	Open label randomized comparison of dihydroartemisinin-piperaquine and artesunate-amodiaquine for the treatment of uncomplicated Plasmodium falciparum malaria in central Vietnam	Tropical medicine & international health : TM & IH	14	5	504-11	19320869
Thapa et al.	2007	Comparison of artemether-lumefantrine with sulfadoxine-pyrimethamine for the treatment of uncomplicated falciparum malaria in eastern Nepal	The American journal of tropical medicine and hygiene	77	3	423-30	17827354
Thriemer et al.	2010	Azithromycin combination therapy for the treatment of uncomplicated falciparum malaria in Bangladesh: an open-label randomized, controlled clinical trial	The Journal of infectious diseases	202	3	392-8	20557237
Thwing et al.	2009	In-vivo efficacy of amodiaquine-artesunate in children with uncomplicated Plasmodium falciparum malaria in western Kenya	Tropical medicine & international health : TM & IH	14	3	294-300	19187521
Tietche et al.	2010	Tolerability and efficacy of a pediatric granule formulation of artesunate-mefloquine in young children from Cameroon with uncomplicated falciparum malaria	The American journal of tropical medicine and hygiene	82	6	1034-40	20519597
Tiono et al.	2009	Chlorproguanil-dapsone-artesunate versus chlorproguanil-dapsone: a randomized, double-blind, phase III trial in African children, adolescents, and adults with uncomplicated Plasmodium falciparum malaria	The American journal of tropical medicine and hygiene	81	6	969-78	19996424
Tjitra et al.	2001	Therapy of uncomplicated falciparum malaria: a randomized trial comparing artesunate plus sulfadoxine-pyrimethamine versus sulfadoxine-pyrimethamine alone in Irian Jaya, Indonesia	The American journal of tropical medicine and hygiene	65	4	309-17	11693875
Toure et al.	2009	A comparative, randomized clinical trial of artemisinin/naphtoquine twice daily one day versus artemether/lumefantrine six doses regimen in children and adults with uncomplicated falciparum malaria in Cote d'Ivoire	Malaria journal	8		148	19575797
Toure et al.	2011	Artesunate/mefloquine paediatric formulation vs. artemether/lumefantrine for the treatment of uncomplicated Plasmodium falciparum in Anonkoua koute, Cote d'Ivoire	Tropical medicine & international health : TM & IH	16	3	290-7	21214690
Trung et al.	2009	A randomized, controlled trial of artemisinin-piperaquine vs dihydroartemisinin-piperaquine phosphate in treatment of falciparum malaria	Chinese journal of integrative medicine	15	3	189-92	19568711
Tshefu et al	2010	Efficacy and safety of a fixed-dose oral combination of pyronaridine-artesunate compared with artemether-lumefantrine in children and adults with uncomplicated Plasmodium falciparum malaria: a randomised non-inferiority trial	Lancet	375	9724	1457-67	20417857
Tun et al.	2009	Efficacy of oral single dose therapy with artemisinin-naphthoquine phosphate in uncomplicated falciparum malaria	Acta tropica	111	3	275-8	19464245
Ursing et al.	2011	Similar efficacy and tolerability of double-dose chloroquine and artemether-lumefantrine for treatment of Plasmodium falciparum infection in Guinea-Bissau: a randomized trial	The Journal of infectious diseases	203	1	109-16	21148503
Valecha et al.	2009	Therapeutic efficacy of artemether-lumefantrine in uncomplicated falciparum malaria in India	Malaria journal	8		107	19454000
Valecha et al.	2010a	Arterolane, a new synthetic trioxolane for treatment of uncomplicated Plasmodium falciparum malaria: a phase II, multicenter, randomized, dose-finding clinical trial	Clinical infectious diseases : an official publication of the Infectious Diseases Society of America	51	6	684-91	20687837
Valecha et al.	2010b	An open-label, randomised study of dihydroartemisinin-piperaquine versus artesunate-mefloquine for falciparum malaria in Asia	PloS one	5	7	e11880	20689583
van den Broek et al.	2006	Efficacy of three artemisinin combination therapies for the treatment of uncomplicated Plasmodium falciparum malaria in the Republic of Congo	Malaria journal	5		113	17125496
van den Broek et al.	2005a	Efficacy of two artemisinin combination therapies for uncomplicated falciparum malaria in children under 5 years, Malakal, Upper Nile, Sudan	Malaria journal	4	1	14	15730557
van den Broek et al.	2005b	Efficacy of chloroquine + sulfadoxine--pyrimethamine, mefloquine + artesunate and artemether + lumefantrine combination therapies to treat Plasmodium falciparum malaria in the Chittagong Hill Tracts, Bangladesh	Transactions of the Royal Society of Tropical Medicine and Hygiene	99	10	727-35	16095643

Additional File 2 : References

Authors	Year	Title	Journal	Volume	Issue	Pages	Accession No.
van Vugt et al.	2000	Artemether-lumefantrine for the treatment of multidrug-resistant falciparum malaria	Transactions of the Royal Society of Tropical Medicine and Hygiene	94	5	545-8	11132386
van Vugt et al.	2002	Treatment of uncomplicated multidrug-resistant falciparum malaria with artesunate-atovaquone-proguanil	Clinical infectious diseases : an official publication of the Infectious Diseases Society of America	35	12	1498-504	12471569
Vasquez et al.	2009	Therapeutic efficacy of a regimen of artesunate-mefloquine-primaquine treatment for Plasmodium falciparum malaria and treatment effects on gametocytic development	Biomedica : revista del Instituto Nacional de Salud	29	2	307-19	20128355
von Seidlein et al.	2000	Efficacy of artesunate plus pyrimethamine-sulphadoxine for uncomplicated malaria in Gambian children: a double-blind, randomised, controlled trial	Lancet	355	9201	352-7	10665554
von Seidlein et al.	2001	Parasitaemia and gametocytaemia after treatment with chloroquine, pyrimethamine/sulfadoxine, and pyrimethamine/sulfadoxine combined with artesunate in young Gambians with uncomplicated malaria	Tropical medicine & international health : TM & IH	6	2	92-8	11251903
Wang et al.	2001	Efficacy of dihydroartemisinin-mefloquine on acute uncomplicated falciparum malaria	Chinese medical journal	114	6	612-3	11780437
Warsame et al.	2009	Efficacy of monotherapies and artesunate-based combination therapies in children with uncomplicated malaria in Somalia	Acta tropica	109	2	146-51	19026606
Wattanakoon et al.	2003	Six-years monitoring the efficacy of the combination of artesunate and mefloquine for the treatment of uncomplicated falciparum malaria	The Southeast Asian journal of tropical medicine and public health	34	3	542-5	15115124
Weerasinghe et al.	2002	A safety and efficacy trial of artesunate, sulphadoxine-pyrimethamine and primaquine in P falciparum malaria	The Ceylon medical journal	47	3	83-5	12449772
Whegang et al.	2010	Efficacy of non-artemisinin- and artemisinin-based combination therapies for uncomplicated falciparum malaria in Cameroon	Malaria journal	9		56	20170477
Wong et al.	2003	Therapeutic equivalence of a low dose artemisinin formulation in falciparum malaria patients	The Journal of pharmacy and pharmacology	55	2	193-8	12631411
Wootton et al.	2008	Open-label comparative clinical study of chlorproguanil-dapsone fixed dose combination (Lapdap) alone or with three different doses of artesunate for uncomplicated Plasmodium falciparum malaria	PloS one	3	3	e1779	18320064
Yavo et al.	2011	Multicentric assessment of the efficacy and tolerability of dihydroartemisinin-piperaquine compared to artemether-lumefantrine in the treatment of uncomplicated Plasmodium falciparum malaria in sub-Saharan Africa	Malaria journal	10		198	21774826
Yeka et al.	2005	Artemisinin versus nonartemisinin combination therapy for uncomplicated malaria: randomized clinical trials from four sites in Uganda	PLoS medicine	2	7	e190	16033307
Yeka et al.	2008	Artemether-lumefantrine versus dihydroartemisinin-piperaquine for treating uncomplicated malaria: a randomized trial to guide policy in Uganda	PloS one	3	6	e2390	18545692
Zongo et al.	2007a	Artemether-lumefantrine versus amodiaquine plus sulfadoxine-pyrimethamine for uncomplicated falciparum malaria in Burkina Faso: a randomised non-inferiority trial	Lancet	369	9560	491-8	17292769
Zongo et al.	2007b	Randomized comparison of amodiaquine plus sulfadoxine-pyrimethamine, artemether-lumefantrine, and dihydroartemisinin-piperaquine for the treatment of uncomplicated Plasmodium falciparum malaria in Burkina Faso	Clinical infectious diseases : an official publication of the Infectious Diseases Society of America	45	11	1453-61	17990228
Zoungrana et al.	2008	Safety and efficacy of methylene blue combined with artesunate or amodiaquine for uncomplicated falciparum malaria: a randomized controlled trial from Burkina Faso	PloS one	3	2	e1630	18286187
