# Supplementary material for: Early parasitological response following artemisinin-containing regimens: a critical review of the literature
Source: Malar J. 2013 Apr 19;12:125. doi: 10.1186/1475-2875-12-125 (PMC3649884; doi:10.1186/1475-2875-12-125)
Supplement: Additional file 4 — Study design of trials included in the analysis. Parasitaemia presented as geometric mean or mean, unless marked with * in which case median. Age presented as mean, unless marked with * in which case median. [file 1475-2875-12-125-S4.pdf]

Additional File 4 : Study Design

Authors	Year	Country	Randomization	Duration of Follow Up (days)	Parasitemia Inclusion Criteria /µL	Parasitemia (µL)	Age Inclusion Criteria	Age (Year)
4ABC Study Group	2011	Burkina Faso; Gabon; Nigeria; Rwanda; Uganda; Zambia; Mozambique	Yes	28	2000-200000	30000*	6-59 months	2.5
Abacassamo et al.	2004	Mozambique	Yes	21	2000-100000	23685*	0.5-5 years	2.5
Abdulla et al.	2008	Benin, Kenya, Mali, Mozambique, and Tanzania	Yes	42	2000-200000	26364*	≤12 years	3*
Achan et al.	2009	Uganda	Yes	28	Any Parasitemia	16124	0.5-5 years	2
Adam et al.	2006	Sudan	No	28	NA	21342	All ages	18
Adam et al.	2005a	Sudan	Yes	28	NA	18257	All ages	15
Adam et al.	2005b	Sudan	Yes	28	NA	15529	All ages	16
Adjei et al.	2008	Ghana	Yes	28	2000-200000	51408	0.5-14 years	5
Adjuik et al.	2002	Kenya, Senegal, and Gabon	Yes	28	1000-200000	26231	0.5-10 years	5.5
Agomo et al.	2008	Nigeria	No	28	1000-250000	6890	Adult and children	22.5
Alecrim et al.	2006	Brazil	Yes	6	1000-50000	NA	≥16 years	35
Allen et al.	2009	Mozambique	Yes	42	up to 500000	2885	>1 years	12*
Asante et al.	2009	Ghana	Yes	28	Any Parasitemia	NA	>14 years	38.1
Ashley et al.	2004	Thailand	Yes	63	NA	9535	1-65 years	21*
Ashley et al.	2005	Thailand	Yes	63	≤4%	10678	1-65 years	19*
Ashley et al.	2006	Thailand	Yes	63	NA	8612	0.5-65 years	20*
Ashley et al.	2007	Thailand	Yes	42	≥80	1981	18-65 years	30*
Asih et al.	2009	Indonesia	Single arm	28	>500	6300*	All ages	29*
Assefa et al.	2010	Ethiopia	Single arm	28	1000-100000	22660	All ages	9
Avila et al.	2004	Bolivia	Yes	28	>250	4445	All ages	28.4
Ayede et al.	2010	Nigeria	Yes	28	2000-200000	11673*	12-156 months	5.1
Barennes et al.	2004	Burkina Faso	Yes	28	>1000	4390	1-15 years	6.6
Bassat et al.	2009	Burkina Faso, Kenya, Mozambique, Uganda and Zambia	Yes	42	2000-200000	25884	0.5-5 years	2.43
Bell et al.	2008	Malawi	Yes	42	2000-200000	36315	12-59 months	4*
Bell et al.	2009	Malawi	Yes	42	NA	21262	≥0.5 years	8.9
Bethell et al.	2011	Cambodia	Yes	42	1000-200000	18266	18-65 years	22*
Blair et al.	2006	Colombia	Yes	28	250-50000	NA	>1 years	NA
Bonnet et al.	2007	Guinea	Yes	28	2000-200000	34716	6-59 months	2.4
Bonnet et al.	2009	The Democratic Republic of Congo	Yes	28	2000-200000	26134	6-59 months	1.8*
Borrmann et al.	2005	Gabon	No	28	1000-100000	34560*	6-12 years	10.3*
Borrmann et al.	2011	Kenya	Yes	63	2000-200000	35600*	6-59 months	2.3*
Bousema et al.	2006	Kenya	Yes	28	≥500	11887	0.5-10 years	4.1*
Bouyou-Akotet et al.	2010	Gabon	Yes	28	1000-250000	36400*	1-13 years	9
Bukirwa et al.	2006	Uganda	Yes	28	2000-200000	22071	1-10 years	1.83*
Campbell et al.	2006	India	Yes	42	150-100000	5157	≥1 years	17*
Chanda et al.	2006	Zambia	Single arm	28	2000-200000	NA	<60 months	1.9
Charle et al.	2009	Equatorial Guinea	Yes	14	2000-200000	24978*	6-59 months	2.2
de Oliveira et al.	2011	Peru	Yes	28	250-100000	3551	≥1 years	22.5*
de Vries et al.	2000	Vietnam	Yes	28	1000-100000	16123	8-65 years	26*
Denis et al.	2002	Cambodia	Single arm	28	>1000	NA	1-60 years	NA
Denis et al.	2006a	Cambodia	Single arm	28	1000-150000	NA	>6 years	NA
Denis et al.	2006b	Cambodia	No	28	1000-150000	16000	>6 years	24
Depoortere et al.	2005	Zambia	Yes	28	2000-100000	19185	6-59 months	1.8
Diem Thuy et al.	2007	Vietnam	Yes	42	1000-200000	NA	≥15 years	28
Djimdé et al.	2008	Mali	Yes	28	2000-200000	15760	≥ 0.5 years	3*
Dondorp et al.	2009	Cambodia and Thailand	Yes	63	≥10000	37214	≥16 years	31
Dunyo et al.	2011	The Gambia	Yes	28	500-200000	21670	6-120 months	5
Durrani et al.	2005	Afghanistan	Yes	42	>1 parasite/HPF	6137	≥3 years	15.3
Elamin et al.	2010	Sudan	Single arm	28	NA	28712	All ages	13
Falade et al.	2005	Kenya, Nigeria, Tanzania	Single arm	28	1000-100000	18488*	Children	2*
Falade et al.	2008a	Nigeria	Yes	28	2000-200000	27822	6-120 months	4.5
Falade et al.	2008b	Nigeria	Single arm	28	1000-100000	26364	Children	3
Fanello et al.	2007	Rwanda	Yes	28	2000-200000	23324	12-59 months	2.8
Fanello et al.	2008	Rwanda	Yes	28	2000-200000	22566	6-59 months	2.4

Additional File 4 : Study Design

Authors	Year	Country	Randomization	Duration of Follow Up (days)	Parasitemia Inclusion Criteria /µL	Parasitemia (µL)	Age Inclusion Criteria	Age (Year)
Faucher et al.	2009	Benin	Yes	42	>1000	49718	6-60 months	2.2
Faye et al.	2007	Senegal	Yes	28	NA	27000	All ages	15
Faye et al.	2010a	Senegal	Yes	28	1000-100000	31677	Children (10-20 kg)	5
Faye et al.	2010b	Senegal and Ivory Coast	Yes	28	1000-200000	30817	>7 years	18
Fehintola et al.	2008	Nigeria	Yes	28	≥ 2000	105426	0.5-10 years	6
Fehintola et al.	2010	Nigeria	Yes	14	≥2000	16596	0.5-12 years	6.1
Gbotosho et al.	2011a	Nigeria	Yes	42	>2000	64145	≤15 years	7.1
Gbotosho et al.	2011b	Nigeria	Yes	42	2000	71794	≤12 years	7.2
Giao et al.	2001	Vietnam	Yes	28	1000-100000	12589	>6 years	25*
Giao et al.	2004	Vietnam	Yes	28	≥1000	19392	>16 years	27*
Gil et al.	2003	Sao Tome and Principe	Yes	28	10000-100000	36579	6-59 months	2.3
Gomez et al.	2003	Ecuador	Yes	28	500-10000	2838	1-12 years	6.7
Grande et al.	2007	Peru	Yes	63	1000-200000	5866	5-60 years	NA
Grandesso et al.	2006	Sierra Leone	Single arm	28	NA	27116	6-59 months	1.4*
Guthmann et al.	2005	Angola	No	28	2000-100000	19460	6-59 months	2.5
Gutman et al.	2009	Peru	Yes	56	250-50000	NA	18 years	36
Hamour et al.	2005	Sudan	Yes	28	2000-200000	22757	6-59 months	2.5
Haque et al.	2007	Bangladesh	Single arm	42	NA	NA	≥ 18 years	22*
Hasugian et al.	2007	Indonesia	Yes	42	<4%	5514	≥1 years	17*
Hatz et al.	2008	Europe and Colombia	Single arm	28	NA	NA	≥18 years	38
Hien et al.	2004	Vietnam	Yes	56	NA	7789	>2 years	26
Hombhanje et al.	2009	Papua New Guinea	Yes	28	≥250	22228	≥14 years	27.5*
Hung et al.	2004	Vietnam	Yes	28	1000-200000	10100	≥6 years	27
Huong et al.	2001	Vietnam	Yes	28	>1000	18200	4-65 years	26.2
Hutagalung et al.	2005	Thailand	Yes	42	NA	8047	All ages	23
Hwang et al.	2011	Ethiopia	Single arm	42	1000-100000	16374	>0.5 years	16.7
Ibrahium et al.	2007	Sudan	No	28	NA	24050	All ages	13
Janssens et al.	2007	Cambodia	Yes	63	<175000	4786	>1 year	21*
Juma et al.	2008	Kenya	Yes	28	2000-200000	34881	6–59 months	2.1*
Kabanywanyi et al.	2007	Tanzania	Yes	28	2000-200000	49348	6-59 years	2
Kamya et al.	2007	Uganda	Yes	42	2000-200000	22789	0.5-10 years	1.5*
Karema et al.	2006	Rwanda	Yes	28	2000-200000	29999	12-59 months	3
Karunajeewa et al.	2003	Papua New Guinea	No	NA	<2000	536*	5-10 years	7.9
Karunajeewa et al.	2008a	Papua New Guinea	Yes	42	>1000	20400	5-10 years	6.9
Karunajeewa et al.	2008b	Papua New Guinea	Yes	42	>1000	50986*	6-60 months	2.9
Kayentao et al.	2009	Mali	Yes	28	2000-200000	24113	6-59 months	2.8
Kobbe et al.	2008	Ghana	Yes	28	2000-200000	38864	6–59 months	2.7
Kofoed et al.	2003	Guinea-Bissau	Yes	35	≥800	19200*	Children	5.2*
Koram et al.	2005	Ghana	Yes	28	2000-200000	19687	6-59 months	2.1*
Koram et al.	2008	Ghana	Single arm	28	2000-200000	35793	6-59 months	2.2*
Krudsood et al.	2000	Thailand	Yes	28	NA	NA	≥14 years	25.7
Krudsood et al.	2002	Thailand	Yes	28	100-400000	NA	Adult and children >25 kg	NA
Krudsood et al.	2003	Thailand	Yes	28	NA	NA	>14 years	28.3
Krudsood et al.	2007	Thailand	Yes	28	NA	8247	≥13 years	NA
Kshirsagar et al.	2000	India	Yes	28	1000-200000	4343*	≥16 years	29*
Lefevre et al.	2001	Thailand	Yes	28	NA	2063	≥12 years	25*
Marquino et al.	2003	Peru	Yes	28	500-30000	6840	5-50 years	25.9
Marquino et al.	2005	Peru	Yes	28	500-50000	7309	≥5 years	28
Martensson et al.	2005	Tanzania	Yes	42	2000-200000	19731	6-59 months	2.0*
Massougbodji et al.	2002	Benin, Cameroon, Ivory Coast	Yes	28	>2000	NA	Adult and children >30 kg	19.5
Mayxay et al.	2004	The Lao PDR	Yes	42	5000-200000	25026	≥1 year	15.4
Mayxay et al.	2006	The Lao PDR	Yes	42	1000-200000	18505	≥1 years	16
Menan et al.	2011	Cameroon, Ivory Coast and Senegal	Yes	28	1000-200000	13225*	≥2 years	15.51
Ménard et al.	2007	Madagascar	Yes	28	1000-200000	22905	0.5-15 years	4
Ménard et al.	2008	Madagascar	Yes	28	1000-200000	14346	0.5-15 years	5*
Mens et al.	2008	Kenya	Yes	28	1000-200000	12145	6-144 months	5*
Meremikwu et al.	2006	Nigeria	Yes	14	1000-200000	7972	6-59 months	2.4

Additional File 4 : Study Design

Authors	Year	Country	Randomization	Duration of Follow Up (days)	Parasitemia Inclusion Criteria /μL	Parasitemia (μL)	Age Inclusion Criteria	Age (Year)
Michael et al.	2010	Nigeria	Yes	42	>2000	78195	1-11 years	6.3
Mockenhaupt et al.	2005	Ghana	Yes	28	2000-200000	35619	6-59 months	2.1*
Mohamed et al.	2006	Sudan	No	28	1760-51600	10215	All ages	23
Mukhtar et al.	2007	Sudan	Yes	28	NA	NA	All ages	18
Mulenga et al.	2006	Zambia	Yes	45	1000	8405	15-50 years	26
Mutabingwa et al.	2005	Tanzania	Yes	28	≥2000	18920*	4-59 months	1.8*
Na-Bangchang et al.	2010	Thailand	Single arm	42	1000-100000	5232*	>15 years	25*
Nahum et al.	2007	Benin	Yes	28	1000-200000	20286	6-59 months	3.1
Nambei et al.	2005	Central Republic of Africa	Yes	14	>600	2700	18-32 years	25
Nambozi et al.	2011	Zambia	Yes	42	2000-200000	35840*	6-59 months	2.4
Ndayiragije et al.	2004	Burundi	No	14	NA	22400	<60 months	3.1
Ndiaye et al.	2008	Senegal and Cameroon	Yes	14	1000-200000	13750*	Any adults or children weighing ≥ 10 kg	NA
Ndiaye et al.	2009	Cameroon, Madagascar, Mali and Senegal	Yes	28	1000-200000	41863	All ages (>= 10 kg)	9.1
Ngasala et al.	2011a	Tanzania	Single arm	42	NA	19054	3-59 months	2.6*
Ngasala et al.	2011b	Tanzania	Yes	56	2000-200000	38272	<60 months	2.8
Nguyen et al.	2003	Vietnam	Yes	28	>1000	4480	4-65 years	10.3
Noedl et al.	2006	Thailand	Yes	28	1000-100000	6761	20-65 years	29
Noedl et al.	2010	Cambodia	Yes	28	100-100000	4227	18-65 years	29
Obonyo et al.	2003	Kenya	Yes	28	≥4000	19858	<5years	1.5
Oduro et al.	2004	Ghana	Yes	14	≥5 parasites/HPF	28200	≥15 years	28.5
Oduro et al.	2008	Ghana	Yes	28	2000-200000	12456	6-120 months	3.7
Osorio et al.	2007	Colombia	Yes	28	250-50000	4416	1-65 years	18.5*
Owusu-Agyei et al.	2008	Ghana	Yes	28	2000-200000	16521	<120 months	3.1
Oyakhirome et al.	2007	Gabon	No	28	NA	7500*	Children	1.3
Penali et al.	2008	Ivory Coast	Yes	28	1000-200000	20112	≥6 years	10
Piola et al.	2005	Uganda	Yes	28	500-100000	NA	All ages	NA
Premji et al.	2009	Burkina Faso, Ghana, Kenya, Nigeria, Tanzania	Yes	42	2000-200000	22383	≥1-<15 years	4
Priotto et al.	2003	Uganda	Yes	28	500-100000	21373	6-59 months	2.4
Pukrittayakamee et al.	2004	Thailand	Yes	28	NA	26566	Adult	24
Rahman et al.	2008	Bangladesh	Yes	42	NA	7019	≥2 years	20
Ramharter et al.	2005	Gabon	Yes	28	1000-100000	12000*	3-12 years	7
Rasheed et al.	2011	Liberia	Single arm	28	NA	NA	>0.5 years	21.76
Ratcliff et al.	2007	Indonesia	Yes	42	<4%	3386	NA	17*
Rojanawatsirivej et al.	2003	Thailand	No	28	NA	5578	Adult	33.03
Rulisa et al.	2007	Rwanda	Yes	28	NA	33182	3-144 months	5.1
Rwagacondo et al.	2003	Rwanda	Yes	28	1000-100000	12667	6-59 months	2.1
Rwagacondo et al.	2004	Rwanda	Yes	28	1000-100000	20522	6-59 months	2.2
Sagara et al.	2006	Mali	Yes	28	1000-100000	13143	≥0.5 years	12
Sagara et al.	2008	Mali	Yes	28	2000-200000	18357	≥1 year	7
Sagara et al.	2009	Cameroon, Mali, Rwanda and Sudan	Yes	28	2000-200000	8400*	> 0.5 years	8*
Schwarz et al.	2005	Gabon	Single arm	28	NA	11600*	Children	0.6
Silachamroon et al.	2005	Thailand	Yes	28	NA	9883	≥15 years	27
Sirima et al.	2003	Burkina Faso	Yes	28	1000-100000	21600*	0.5-5 years	1.2
Sirima et al.	2009	Burkina Faso	Yes	28	>1000	NA	6-59 months	2.3
Sirivichayakul et al.	2007	Thailand	Yes	28	500-150000	27114	1-14 years	8.3
Smithuis et al.	2006	Myanmar	Yes	42	500-100000	8128	≥1 years	NA
Smithuis et al.	2010	Myanmar	Yes	63	500-200000	7849	>0.5 years	NA
Smithuis et al.	2004a	Myanmar	Yes	42	500-100000	6026	≥1 years	NA
Smithuis et al.	2004b	Myanmar	Yes	42	1000-250000	11609	All ages	16.7
Song et al.	2011	Cambodia	Yes	28	1000-100000	26637	7-65 years	25.2
Sowunmi et al.	2005	Nigeria	Yes	28	>2000	32173	≤12 years	6.7
Sowunmi et al.	2009	Nigeria	Yes	42	>2000	30654	≤120 months	6.2
Sowunmi et al.	2011	Nigeria	Yes	42	2000	64165	≤12 years	7.5
Sowunmi et al.	2007a	Nigeria	Yes	35	>2000	47627	<11 years	5.9

Additional File 4 : Study Design

Authors	Year	Country	Randomization	Duration of Follow Up (days)	Parasitemia Inclusion Criteria /µL	Parasitemia (µL)	Age Inclusion Criteria	Age (Year)
Sowunmi et al.	2007b	Nigeria	Yes	42	>2000	46541	≤120 months	5.5
Staedke et al.	2004	Uganda	Yes	28	500-200000	22483	0.5-10 years	4*
Stohrer et al.	2004	The Lao PDR	Yes	42	1000-100000	6400	All ages	9*
Suputtamongkol et al.	2003	Thailand	Yes	42	NA	NA	All ages	NA
Sutherland et al.	2003	The Gambia	Yes	28	>500	NA	1-9 years	4.9
Sutherland et al.	2005	The Gambia	Yes	28	500-250000	56689	1-10 years	4
Swarthout et al.	2006	The Democratic Republic of Congo	Yes	28	2000-200000	27392	6-59 months	2
Sykes et al.	2009	Tanzania	Yes	42	2000-200000	20960*	6-59 months	2.5*
Tall et al.	2007	The Comoros Union	Yes	14	1000-200000	4788	2-70 years	6*
Tangpukdee et al.	2005	Thailand	Yes	28	NA	4645	>14 years	26.5
Tangpukdee et al.	2008	Thailand	Yes	28	NA	12585	≥15 years	22*
Thanh et al.	2009	Vietnam	Yes	42	200-200000	14074	5-60 years	23.5
Thapa et al.	2007	Nepal	Yes	28	>500	6492*	>5 years	26
Thriemer et al.	2010	Bangladesh	Yes	42	100-100000	9781	8-65 years	22
Thwing et al.	2009	Kenya	Single arm	28	2000-200000	54915	6-59 months	2.6
Tietche et al.	2010	Cameroon	Single arm	63	1000-250000	20040*	Children (10-20 kg)	3
Tiono et al.	2009	Burkina Faso, Ghana, Mali, Nigeria	Yes	28	2000-200000	21640	≥1 years	7.4
Tjitra et al.	2001	Indonesia	Yes	28	1000-100000	1960	≥0.5 years	9.5
Toure et al.	2009	Ivory Coast	Yes	28	2000-200000	10186	≥6 months	NA
Toure et al.	2011	Ivory coast	Yes	28	2000-200000	19685	6-59 months	NA
Trung et al.	2009	Vietnam	Yes	28	1000-100000	20428*	7-65 years	25.8*
Tshefu et al	2010	The Democratic Republic of Congo; The	Yes	42	1000–100000	14813	3-60 years	18
Tun et al.	2009	Myanmar	Single arm	28	1000-100000	17556	15-55 years	27
Ursing et al.	2011	Guinea-Bissau	Yes	42	800-200000	22346*	6-180 months	6.5*
Valecha et al.	2009	India	Single arm	28	1000-100000	7653	Adult and children >5 kg	11.1
Valecha et al.	2010a	Thailand, India, and Tanzania	Yes	28	1000-100000	NA	13-65 years	26.4
Valecha et al.	2010b	Thailand, The Lao PDR and India	Yes	63	80-200000	7924	0.25-65 years	25.4
van den Broek et al.	2006	The Democratic Republic of Congo	Yes	28	2000-200000	24997	6-59 months	1.9
van den Broek et al.	2005a	Sudan	Yes	42	2000-200000	24835	6-59 months	2.8
van den Broek et al.	2005b	Bangladesh	Yes	42	1000-100000	11814	≥1 years	15.7
van Vugt et al.	2000	Thailand	Yes	28	≥500	NA	All ages	NA
van Vugt et al.	2002	Thailand	Yes	42	NA	4211	Adult and children	22.3
Vasquez et al.	2009	Colombia	Yes	42	150-50000	3280*	>1 years	NA
von Seidlein et al.	2000	The Gambia	Yes	28	≥500	19000*	0.5-10 years	4.83
von Seidlein et al.	2001	The Gambia	Yes	28	>500	9080	<18 years	6.6
Wang et al.	2001	Equatorial Guinea	Single arm	28	NA	15217	Adult and children	25.5
Warsame et al.	2009	Somalia	No	28	2000-200000	NA	6-120 months	NA
Wattanakoon et al.	2003	Thailand	Single arm	42	NA	NA	Adult	NA
Weerasinghe et al.	2002	Sri Lanka	Single arm	28	NA	NA	>15 years	NA
Whegang et al.	2010	Cameroon	Yes	28	>2000	40170	<60 months	2.4
Wong et al.	2003	Malaysia	Yes	35	200-100000	7798	16-61 years	38
Wootton et al.	2008	Malawi and The Gambia	Yes	14	10000-100000	NA	1-60 years	NA
Yavo et al.	2011	Cameroon, Ivory Coast and Senegal	Yes	28	1000-200000	10840*	≥2 years	15.64
Yeka et al.	2005	Uganda	Yes	28	2000-200000	NA	≥6 months	3.5*
Yeka et al.	2008	Uganda	Yes	42	2000-200000	35211	0.5-10 years	2*
Zongo et al.	2007a	Burkina Faso	Yes	28	NA	27689	≥0.5 years	4*
Zongo et al.	2007b	Burkina Faso	Yes	42	2000-200000	27110	≥0.5 years	4*
Zoungrana et al.	2008	Burkina Faso	Yes	28	≥1000	31000*	6–10 years	7*
