# Supplementary material for: Early parasitological response following artemisinin-containing regimens: a critical review of the literature
Source: Malar J. 2013 Apr 19;12:125. doi: 10.1186/1475-2875-12-125 (PMC3649884; doi:10.1186/1475-2875-12-125)
Supplement: Additional file 5 — Parasite positivity rates and covariates. Red font denotes zero positivity rate extrapolated from previous recording of parasitaemia or derived from published figures rather than text. [file 1475-2875-12-125-S5.pdf]

Additional File 5 : Parasite Prevalence Rates

Authors	Year	Country	Artemisinin Derivatives	Partner Drug	Artemisinin Derivatives Dose (mg/kg/day)	Artemisinin Derivatives Dose (mg/day)	Doses Per Day	Days of Artemisinin	Supervised	Sample Size	D1 Parasitemic (%)	D2 Parasitemic (%)	D3 Parasitemic (%)	Comments
4ABC Study Group	2011	Burkina Faso; Gabon; Nigeria; Rw	Artesunate	Amodiaquine	2.8-5.5		1	3	Yes	1002				
4ABC Study Group	2011	Burkina Faso; Gabon; Nigeria; Rw	Artesunate	Chlorproguanil+Dapsone	3.4-5		1	3	Yes	413				
4ABC Study Group	2011	Burkina Faso; Gabon; Nigeria; Rw	Artemether	Lumefantrine	3.5-8		2	3	Yes	1226				
4ABC Study Group	2011	Burkina Faso; Gabon; Nigeria; Rw	Dihydroartemisinin	Piperaquine	2.25		1	3	Yes	1475				
Abacassamo et al.	2004	Mozambique	Artesunate	Amodiaquine	4		1	3	Yes	53				
Abacassamo et al.	2004	Mozambique	Artesunate	Sulfadoxine+Pyrimethamine	4		1	3	Yes	53				
Abdulla et al.	2008	Benin, Kenya, Mali, Mozambique	Artemether	Lumefantrine	3.5-8		2	3	Yes	447	61.5	11.5	0.7	Dispersible tablet
Abdulla et al.	2008	Benin, Kenya, Mali, Mozambique	Artemether	Lumefantrine	3.5-8		2	3	Yes	452	62.6	10.6	0.7	Crushed tablet
Achan et al.	2009	Uganda	Artemether	Lumefantrine	4-4.6		2	3	Partial	89				
Adam et al.	2005a	Sudan	Artesunate	Mefloquine	4		1	3	Yes	38				
Adam et al.	2005b	Sudan	Artesunate	Sulfadoxine+Pyrimethamine	4		1	3	Yes	30	6.7		0	
Adam et al.	2006	Sudan	Artesunate	Sulfamethoxypyrazine+Pyrimethamine		50-400	1-2	2	Yes	39	2.6		0	Fixed Dose
Adam et al.	2006	Sudan	Artesunate	Sulfamethoxypyrazine+Pyrimethamine		50-200	1	3	Yes	34			0	Loose
Adjei et al.	2008	Ghana	Artesunate	Amodiaquine	4		1	3	Yes	116	50	10	0	PPR derived from figure
Adjei et al.	2008	Ghana	Artemether	Lumefantrine	4.6-8		2	3	Partial	111	50	8	0	PPR derived from figure
Adjuik et al.	2002	Kenya, Senegal, and Gabon	Artesunate	Amodiaquine	4		1	3	Yes	160	80	35	10	PPR derived from figure
Adjuik et al.	2002	Kenya, Senegal, and Gabon	Artesunate	Amodiaquine	4		1	3	Yes	200	80	16	6	PPR derived from figure
Adjuik et al.	2002	Kenya, Senegal, and Gabon	Artesunate	Amodiaquine	4		1	3	Yes	110	50	10	5	PPR derived from figure
Agomo et al.	2008	Nigeria	Artesunate	Mefloquine	4		1	3	Yes	208	59.1	7.9	0	Adult
Agomo et al.	2008	Nigeria	Artesunate	Mefloquine	4		1	3	Yes	238	68.4	8.3	0	Child
Alecrim et al.	2006	Brazil	Artemether	Lumefantrine		160	2	3	Yes	28	76	17.9	0	
Allen et al.	2009	Mozambique	Artesunate	Sulfadoxine+Pyrimethamine	4		1	3	Yes	212				
Asante et al.	2009	Ghana	Artesunate	Amodiaquine	3		2	3	Partial	190				Unsupervised
Asante et al.	2009	Ghana	Artesunate	Amodiaquine	3.1		2	3	Yes	211				
Ashley et al.	2007	Thailand	Artemether	Lumefantrine	Not specified		2	3	Yes	17			0	
Ashley et al.	2007	Thailand	Artemether	Lumefantrine	Not specified		1	3	Yes	19			0	
Ashley et al.	2004	Thailand	Artesunate	Mefloquine	4		1	3	Yes	177	81.8	21.6	2.8	
Ashley et al.	2004	Thailand	Artesunate	Mefloquine	4		1	3	Yes	67				
Ashley et al.	2005	Thailand	Artesunate	Mefloquine	4		1	3	Yes	166	84	17	2	
Ashley et al.	2006	Thailand	Artesunate	Mefloquine	4		1	3	Yes	249	78	29	6	Loose
Ashley et al.	2006	Thailand	Artesunate	Mefloquine	4.17		1	3	Yes	251	84	39	8	Fixed Dose
Ashley et al.	2005	Thailand	Dihydroartemisinin	Piperaquine	2.1		1	3	Yes	170	79	19	2	
Ashley et al.	2004	Thailand	Dihydroartemisinin	Piperaquine	1.5-3.0		1-2	3	Yes	67				
Ashley et al.	2004	Thailand	Dihydroartemisinin	Piperaquine	1.6-3.2		1-2	3	Yes	179	70.9	17.9	2.2	
Ashley et al.	2005	Thailand	Dihydroartemisinin	Piperaquine	1.6-3.2		1-2	3	Yes	163	74	20	2	
Ashley et al.	2004	Thailand	Dihydroartemisinin	Piperaquine+Artesunate	4		1-2	3	Yes	174	67.2	8.6	1.1	
Ashley et al.	2004	Thailand	Dihydroartemisinin	Piperaquine+Artesunate	1.5-3.0		1-2	3	Yes	67				
Asih et al.	2009	Indonesia	Artesunate	Amodiaquine	4		1	3	Yes	103			12.6	
Assefa et al.	2010	Ethiopia	Artemether	Lumefantrine	Not specified		2	3	Yes	90			1.1	
Avila et al.	2004	Bolivia	Artesunate	Mefloquine	4		1	3	Yes	70		21.4	8.5	
Ayede et al.	2010	Nigeria	Artesunate	Amodiaquine		50-100	1	3	Yes	250	29.4	3.8	0	Fixed Dose
Ayede et al.	2010	Nigeria	Artesunate	Sulphamethoxypyrazine+Pyrimethamine		50-200	1-2	2	Yes	250	28.8	5.3	1.3	Fixed Dose
Barennes et al.	2004	Burkina Faso	Artesunate	Amodiaquine	4		1	3	Yes	33	15.2	0	0	
Barennes et al.	2004	Burkina Faso	Artesunate		2-4		1	3	Yes	27	11	0	0	
Bassat et al.	2009	Burkina Faso, Kenya, Mozambiqu	Artemether	Lumefantrine	4.6-8		2	3	Yes	510				
Bassat et al.	2009	Burkina Faso, Kenya, Mozambiqu	Dihydroartemisinin	Piperaquine	2.25		1	3	Yes	1038				
Bell et al.	2009	Malawi	Artemether	Lumefantrine	4-4.6		2	3	Partial	209				
Bell et al.	2008	Malawi	Artesunate	Sulfadoxine+Pyrimethamine	4		1	3	Yes	114		5		
Bethell et al.	2011	Cambodia	Artesunate		2		1	7	Yes	75			49	
Bethell et al.	2011	Cambodia	Artesunate		4		1	7	Yes	40			46	
Bethell et al.	2011	Cambodia	Artesunate		6		1	7	Yes	28			48	
Blair et al.	2006	Colombia	Artesunate	Sulfadoxine+Pyrimethamine	4		1	3	Yes	57				
Bonnet et al.	2007	Guinea	Artesunate	Amodiaquine	4		1	3	Yes	110			4.6	
Bonnet et al.	2009	The Democratic Republic of Cong	Artesunate	Amodiaquine	4		1	3	Yes	58		15.5		
Bonnet et al.	2009	The Democratic Republic of Cong	Artesunate	Amodiaquine	4		1	3	Yes	90		64.4		
Bonnet et al.	2007	Guinea	Artesunate	Sulfadoxine+Pyrimethamine	4		1	3	Yes	110			3.7	
Bonnet et al.	2009	The Democratic Republic of Cong	Artesunate	Sulfadoxine+Pyrimethamine	4		1	3	Yes	62		12.9		
Bonnet et al.	2009	The Democratic Republic of Cong	Artesunate	Sulfadoxine+Pyrimethamine	4		1	3	Yes	91		65.9		
Borrmann et al.	2005	Gabon	Artesunate	Fosmidomycin	4		2	3	Yes	10				

Additional File 5 : Parasite Prevalence Rates

Authors	Year	Country	Artemisinin Derivatives	Partner Drug	Artemisinin Derivatives Dose (mg/kg/day)	Artemisinin Derivatives Dose (mg/day)	Doses Per Day	Days of Artemisinin	Supervised	Sample Size	D1 Parasitemic (%)	D2 Parasitemic (%)	D3 Parasitemic (%)	Comments
Borrmann et al.	2005	Gabon	Artesunate	Fosmidomycin	4		2	1	Yes	10				
Borrmann et al.	2005	Gabon	Artesunate	Fosmidomycin	4		2	2	Yes	10				
Borrmann et al.	2005	Gabon	Artesunate	Fosmidomycin	2-4		2	4	Yes	10				
Borrmann et al.	2005	Gabon	Artesunate	Fosmidomycin	2-4		2	5	Yes	10				
Borrmann et al.	2011	Kenya	Artemether	Lumefantrine	4		2	3	Yes	241	90.3	11	0	
Borrmann et al.	2011	Kenya	Dihydroartemisinin	Piperaquine	2.25		1	3	Yes	233	66.8	4.4	0	
Bousema et al.	2006	Kenya	Artemether	Lumefantrine	Not specified		2	3	Yes	75				
Bousema et al.	2006	Kenya	Artesunate	Sulfadoxine+Pyrimethamine	4		1	3	Yes	174				
Bouyou-Akotet et al.	2010	Gabon	Artesunate	Mefloquine	4		1	3	Yes	30				
Bouyou-Akotet et al.	2010	Gabon	Artesunate	Mefloquine	4		1	3	Yes	41				pediatric group
Bukirwa et al.	2006	Uganda	Artesunate	Amodiaquine	4		1	3	Yes	201		2.5	0.5	
Bukirwa et al.	2006	Uganda	Artemether	Lumefantrine	4-4.6		2	3	Yes	202		2	0.5	
Campbell et al.	2006	India	Artesunate	Mefloquine	4		1	3	Yes	58		11		
Campbell et al.	2006	India	Artesunate	Mefloquine	4		1	3	Yes	58		12		
Chanda et al.	2006	Zambia	Artemether	Lumefantrine	Not specified		1	3	Yes	111				
Charle et al.	2009	Equatorial Guinea	Artesunate	Sulfadoxine+Pyrimethamine	4		1	3	Yes	86	77.2	3.8	0	
de Oliveira et al.	2011	Peru	Artesunate	Mefloquine	4		1	3	Partial	96			0	Unsupervised
de Oliveira et al.	2011	Peru	Artesunate	Mefloquine	4		1	3	Yes	96			0	
de Vries et al.	2000	Vietnam	Artemisinin	Quinine	20		1	1	Yes	88				
de Vries et al.	2000	Vietnam	Artemisinin	Quinine	20		1	1	Yes	96				
Denis et al.	2006b	Cambodia	Artemether	Lumefantrine	4.6-5		2	3	Yes	80				
Denis et al.	2006b	Cambodia	Artemether	Lumefantrine	4.6-5		2	3	Yes	55				
Denis et al.	2006a	Cambodia	Artesunate	Mefloquine	4		1	3	Yes	255				
Denis et al.	2006a	Cambodia	Artesunate	Mefloquine	4		1	3	Yes	1025				
Denis et al.	2006b	Cambodia	Artesunate	Mefloquine	4		1	3	Yes	55				
Denis et al.	2002	Cambodia	Dihydroartemisinin	Piperaquine	3.3-4.6		2	2	Yes	106			0	
Depoortere et al.	2005	Zambia	Artesunate	Sulfadoxine+Pyrimethamine	4		1	3	No	84				Unsupervised
Depoortere et al.	2005	Zambia	Artesunate	Sulfadoxine+Pyrimethamine	4		1	3	Yes	85				Supervised
Diem Thuy et al.	2007	Vietnam	Dihydroartemisinin	Mefloquine		300	1	2	Yes	44				
Diem Thuy et al.	2007	Vietnam	Dihydroartemisinin			100-300	1	5	Yes	45				
Djimdé et al.	2008	Mali	Artesunate	Amodiaquine	4		1	3	Yes	252			0	PPR derived from figure
Djimdé et al.	2008	Mali	Artesunate	Sulfadoxine+Pyrimethamine	4		1	3	Yes	250			0	PPR derived from figure
Djimdé et al.	2008	Mali	Artesunate		2-4		1	5	Yes	251			0	PPR derived from figure
Dondorp et al.	2009	Cambodia and Thailand	Artesunate	Mefloquine	4		1	3	Yes	20		73	55	
Dondorp et al.	2009	Cambodia and Thailand	Artesunate	Mefloquine	4		1	3	Yes	20		45	8	
Dondorp et al.	2009	Cambodia and Thailand	Artesunate		2		1	7	Yes	20		73	55	
Dondorp et al.	2009	Cambodia and Thailand	Artesunate		2		1	7	Yes	20		45	8	
Dunyo et al.	2011	The Gambia	Artemether	Lumefantrine	4-4.6		2	3	Partial	618				
Durrani et al.	2005	Afghanistan	Artesunate	Amodiaquine	4		1	3	Yes	79	37.5	4	4	PPR derived from figure
Elamin et al.	2010	Sudan	Artemether	Lumefantrine	Not specified		2	3	Yes	291	24.4		8.7	
Falade et al.	2008a	Nigeria	Artesunate	Amodiaquine	4		1	3	Yes	66				
Falade et al.	2005	Kenya, Nigeria, Tanzania	Artemether	Lumefantrine	3.2-8		2	3	Yes	310	44.3	1.6		
Falade et al.	2008b	Nigeria	Artemether	Lumefantrine	3.2-8		2	3	Yes	103	24.7		0	
Falade et al.	2008a	Nigeria	Artemether	Lumefantrine	3.5-8		2	3	Yes	66				
Fanello et al.	2008	Rwanda	Artesunate	Chlorproguanil+Dapsone	4		1	3	Yes	400		1.3	0	
Fanello et al.	2007	Rwanda	Artemether	Lumefantrine	3.3-8		2	3	Yes	251		2.8	0	
Faucher et al.	2009	Benin	Artesunate	Amodiaquine	5-5.6		1	3	Yes	96				
Faucher et al.	2009	Benin	Artemether	Lumefantrine	4-8		2	3	Yes	96				
Faye et al.	2007	Senegal	Artesunate	Amodiaquine	4		1	3	Yes	360				
Faye et al.	2010b	Senegal and Ivory Coast	Artesunate	Amodiaquine	2.32-4.65		1	3	Yes	159		0	0	
Faye et al.	2010a	Senegal	Artemether	Lumefantrine	4		2	3	Yes	160		1.9		
Faye et al.	2007	Senegal	Artemether	Lumefantrine	Not specified		1-2	2	Yes	140				
Faye et al.	2007	Senegal	Artemether	Lumefantrine	Not specified		2	3	Yes	149				
Faye et al.	2010b	Senegal and Ivory Coast	Artemether	Lumefantrine	Not specified		2	3	Yes	163		0	0	
Faye et al.	2010a	Senegal	Artesunate	Mefloquine	2.5-5		1	3	Yes	160		2.5		
Faye et al.	2007	Senegal	Artesunate	Mefloquine		100-200	1	3	Yes	145				
Fehintola et al.	2008	Nigeria	Artesunate	Amodiaquine	4		1	3	Yes	61	70	14.7		
Fehintola et al.	2010	Nigeria	Artesunate	Chloroquine	4		2	3	Yes	26	50	7.7	0	
Fehintola et al.	2008	Nigeria	Artesunate	Cotrimoxazole	4		1	3	Yes	121	30	0.8		

Additional File 5 : Parasite Prevalence Rates

Authors	Year	Country	Artemisinin Derivatives	Partner Drug	Artemisinin Derivatives Dose (mg/kg/day)	Artemisinin Derivatives Dose (mg/day)	Doses Per Day	Days of Artemisinin	Supervised	Sample Size	D1 Parasitemic (%)	D2 Parasitemic (%)	D3 Parasitemic (%)	Comments
Fehintola et al.	2010	Nigeria	Artesunate	Cotrimoxazole	4		2	3	Yes	31	35.5	0	0	
Gbotosho et al.	2011b	Nigeria	Artesunate	Amodiaquine	4		1	3	Yes	104	22.1			
Gbotosho et al.	2011a	Nigeria	Artesunate	Amodiaquine	5.5		1	3	Yes	100	6			Coformulated
Gbotosho et al.	2011a	Nigeria	Artesunate	Amodiaquine	4.4-10		1	3	Yes	104	5.8			Copackaged
Gbotosho et al.	2011b	Nigeria	Artesunate	Amodiaquine	4.4-10		1	3	Yes	27	3.7			
Gbotosho et al.	2011b	Nigeria	Artesunate	Amodiaquine	4.4-10		1	3	Yes	104	6.7			
Gbotosho et al.	2011b	Nigeria	Artemether	Lumefantrine	5.6		2	3	Yes	33	3			
Gbotosho et al.	2011a	Nigeria	Artemether	Lumefantrine	4.6-8		2	3	Yes	81	4.9			
Gbotosho et al.	2011b	Nigeria	Artemether	Lumefantrine	4.6-8		2	3	Yes	36	11.1			
Giao et al.	2004	Vietnam	Dihydroartemisinin	Piperaquine+Trimethoprim+Primaquine		64-128	1-2	3	Yes	82				
Giao et al.	2001	Vietnam	Artemisinin		10.2		1-2	5	Yes	115				
Giao et al.	2001	Vietnam	Artemisinin		10.3		1-2	7	Yes	112				
Gil et al.	2003	Sao Tome and Principe	Artesunate	Chloroquine	4		1	3	Yes	200	74.4	14.9	3.1	
Gomez et al.	2003	Ecuador	Artesunate	Mefloquine	6.6-13.8		1-3	3	Yes	50				Rectal
Gomez et al.	2003	Ecuador	Artesunate	Mefloquine	6.6-13.8		1-3	3	Yes	50				Rectal
Gomez et al.	2003	Ecuador	Artesunate		3.9-9.5		1-3	6	Yes	50				Rectal
Grande et al.	2007	Peru	Artesunate	Mefloquine	4		1	3	Yes	260	41.5	3.8	0	
Grande et al.	2007	Peru	Dihydroartemisinin	Piperaquine	2.1		1	3	Yes	262	32.3	1.1	0	
Grandesso et al.	2006	Sierra Leone	Artesunate	Amodiaquine	4		1	3	Yes	126			5.8	
Guthmann et al.	2005	Angola	Artesunate	Amodiaquine	4		1	3	Yes	97				
Guthmann et al.	2005	Angola	Artesunate	Sulfadoxine+Pyrimethamine	4		1	3	Yes	90				
Gutman et al.	2009	Peru	Artesunate	Mefloquine	4		1	3	Yes	39			2.6	
Hamour et al.	2005	Sudan	Artesunate	Amodiaquine	4		1	3	Yes	80			1.1	
Hamour et al.	2005	Sudan	Artesunate	Sulfadoxine+Pyrimethamine	4		1	3	Yes	81			1.2	
Haque et al.	2007	Bangladesh	Artemether	Lumefantrine		160	2	3	Yes	67	16.42			
Hasugian et al.	2007	Indonesia	Artesunate	Amodiaquine	4		1	3	Yes	166		0	0	
Hasugian et al.	2007	Indonesia	Dihydroartemisinin	Piperaquine	2.25		1	3	Yes	168		0	0	
Hatz et al.	2008	Europe and Colombia	Artemether	Lumefantrine		160	2	3	Not stated	165	76.2	50.8		
Hien et al.	2004	Vietnam	Artesunate	Mefloquine	4		1	3	Yes	77		22		
Hien et al.	2004	Vietnam	Artesunate	Mefloquine	4		1	3	Yes	38				
Hien et al.	2004	Vietnam	Dihydroartemisinin	Piperaquine	1.7-3.4		1-2	3	Yes	166		27		
Hien et al.	2004	Vietnam	Dihydroartemisinin	Trimethoprim+Piperaquine	1.2-2.4		1-2	3	Yes	76				
Hien et al.	2004	Vietnam	Dihydroartemisinin	Trimethoprim+Piperaquine	1.33-2.66		1-2	3	Yes	157		32		
Hombhanje et al.	2009	Papua New Guinea	Artemisinin	Naphthoquine		1000	1	1	Yes	51	51	8	2	PPR derived from figure
Hung et al.	2004	Vietnam	Artesunate	Mefloquine	4		1	1	Yes	110				
Hung et al.	2004	Vietnam	Artesunate	Mefloquine	4		1	1	Yes	113				
Hung et al.	2004	Vietnam	Artesunate	Mefloquine	4		1	1	Yes	114				
Huong et al.	2001	Vietnam	Artesunate		2-4		1	5	Yes	25				
Hutagalung et al.	2005	Thailand	Artemether	Lumefantrine	2.7-4.6		2	3	Yes	245	73.6	13.2	0.8	
Hutagalung et al.	2005	Thailand	Artesunate	Mefloquine	4		1	3	Yes	245	66.9	14	1.7	
Hwang et al.	2011	Ethiopia	Artemether	Lumefantrine	4.6-8		2	3	Partial	120		6.9	0.9	
Ibrahium et al.	2007	Sudan	Artesunate	Amodiaquine	4		1	3	Yes	42	31		2.4	
Ibrahium et al.	2007	Sudan	Artesunate	Sulfadoxine+Pyrimethamine	4		1	3	Yes	40	20		0	
Janssens et al.	2007	Cambodia	Artesunate	Mefloquine	4		1	3	Yes	236	51	6		
Janssens et al.	2007	Cambodia	Dihydroartemisinin	Piperaquine	1.6-3.2		1-2	3	Yes	228	38	6		
Juma et al.	2008	Kenya	Artemether	Lumefantrine	Not specified		1	3	Yes	121	95	36	0	Suspension; PPR derived from figure
Juma et al.	2008	Kenya	Artemether	Lumefantrine	Not specified		2	3	Yes	124	95	38	0	PPR derived from figure
Kabanywanyi et al.	2007	Tanzania	Artesunate	Amodiaquine	4		1	3	Yes	76				
Kabanywanyi et al.	2007	Tanzania	Artemether	Lumefantrine	3.3-8		2	3	Yes	99				
Kamya et al.	2007	Uganda	Artemether	Lumefantrine	4.6-8		2	3	Yes	210		1	0	
Kamya et al.	2007	Uganda	Dihydroartemisinin	Piperaquine	2.1		1	3	Yes	211		0.5	0	
Karema et al.	2006	Rwanda	Artesunate	Amodiaquine	4		1	3	Yes	252		11.2	0.8	
Karema et al.	2006	Rwanda	Dihydroartemisinin	Piperaquine	1.6-3.1		1	3	Yes	252		6	0.4	
Karunajeewa et al.	2008b	Papua New Guinea	Artemether	Lumefantrine	3.4		2	3	Partial	127				
Karunajeewa et al.	2008a	Papua New Guinea	Dihydroartemisinin	Piperaquine	2.5		1	3	Yes	22				
Karunajeewa et al.	2008b	Papua New Guinea	Dihydroartemisinin	Piperaquine	2.5		1	3	Yes	123				
Karunajeewa et al.	2008b	Papua New Guinea	Artesunate	Sulfadoxine+Pyrimethamine	4		1	3	Yes	122				
Karunajeewa et al.	2003	Papua New Guinea	Artesunate		12.5 +/- 0.7		2	1	Yes	12	0	0	0	Rectal
Karunajeewa et al.	2003	Papua New Guinea	Artesunate		12.9 +/- 0.9		2	1	Yes	30	3.3			Rectal

Additional File 5 : Parasite Prevalence Rates

Authors	Year	Country	Artemisinin Derivatives	Partner Drug	Artemisinin Derivatives Dose (mg/kg/day)	Artemisinin Derivatives Dose (mg/day)	Doses Per Day	Days of Artemisinin	Supervised	Sample Size	D1 Parasitemic (%)	D2 Parasitemic (%)	D3 Parasitemic (%)	Comments
Kayentao et al.	2009	Mali	Artesunate	Amodiaquine	4		1	3	Yes	133		10.5	1.5	
Kayentao et al.	2009	Mali	Artesunate	Sulfadoxine+Pyrimethamine	4		1	3	Yes	132		10.7	0.8	
Kobbe et al.	2008	Ghana	Artesunate	Amodiaquine		25-100	1	3	Partial	123				
Kobbe et al.	2008	Ghana	Artemether	Lumefantrine	3.5-8		2	3	Partial	123				
Kofoed et al.	2003	Guinea-Bissau	Artesunate	Chloroquine	2-4		1-2	3	Yes	119		10		
Kofoed et al.	2003	Guinea-Bissau	Artesunate	Chloroquine	2-4		1-2	3	Yes	120		11		Sequential
Kofoed et al.	2003	Guinea-Bissau	Artesunate		2-4		1-2	3	Yes	115		6		
Koram et al.	2005	Ghana	Artesunate	Amodiaquine	4		1	3	Yes	54	40	2	0	PPR derived from figure
Koram et al.	2008	Ghana	Artesunate	Amodiaquine	4		1	3	Yes	545				
Koram et al.	2005	Ghana	Artemether	Lumefantrine	Not specified		2	3	Yes	51	64	0	0	PPR derived from figure
Krudsood et al.	2000	Thailand	Artesunate	Azithromycin		200	1	3	Yes	67				
Krudsood et al.	2007	Thailand	Artemether	Lumefantrine	3.2		2	3	Yes	94				
Krudsood et al.	2003	Thailand	Artemether	Lumefantrine	4.6		2	3	Yes	41				
Krudsood et al.	2007	Thailand	Artesunate	Mefloquine	4		1	3	Yes	27				
Krudsood et al.	2002	Thailand	Artesunate	Mefloquine	4-5		1	3	Yes	102				Sequential
Krudsood et al.	2002	Thailand	Artesunate	Mefloquine	4-5		1	3	Yes	102				Simultaneous
Krudsood et al.	2000	Thailand	Artesunate	Mefloquine		200	1	3	Yes	67				
Krudsood et al.	2003	Thailand	Dihydroartemisinin	Naphthoquine+Trimethoprim		320	2	1	Yes	89				
Krudsood et al.	2007	Thailand	Dihydroartemisinin	Piperaquine	2		1	3	Yes	97				
Krudsood et al.	2007	Thailand	Artemisinin	Piperaquine	2.4		1	2	Yes	17				
Krudsood et al.	2007	Thailand	Artemisinin	Piperaquine	2.5		1	2	Yes	78				
Krudsood et al.	2007	Thailand	Artemisinin	Piperaquine	3.2		1	3	Yes	61				
Krudsood et al.	2007	Thailand	Artemisinin	Piperaquine	3.2-6.4		1-2	2	Yes	37				
Krudsood et al.	2000	Thailand	Artesunate			200	1	3	Yes	68				
Kshirsagar et al.	2000	India	Artemether	Lumefantrine		20-60	1-3	3	Yes	89				
Lefevre et al.	2001	Thailand	Artemether	Lumefantrine	4.6		2	3	Yes	164	60	5	0	
Lefevre et al.	2001	Thailand	Artesunate	Mefloquine	4		1	3	Yes	55	60	5	0	
Marquino et al.	2003	Peru	Artesunate	Mefloquine	4		1	3	Yes	51		17.7	2	
Marquino et al.	2005	Peru	Artesunate	Sulfadoxine+Pyrimethamine	4		1	3	Yes	94			2.2	
Martensson et al.	2005	Tanzania	Artesunate	Amodiaquine	4		1	3	Yes	207	66	10	0	
Martensson et al.	2005	Tanzania	Artemether	Lumefantrine	3.2-4.4		2	3	Yes	200	83	10	0	
Massougbodji et al.	2002	Benin, Cameroon, Ivory Coast	Artesunate	Mefloquine	4.27		1	3	Yes	52		34		Simultaneous
Massougbodji et al.	2002	Benin, Cameroon, Ivory Coast	Artesunate	Mefloquine	4.43		1	3	Yes	52		35		Sequential
Mayxay et al.	2004	The Lao PDR	Artemether	Lumefantrine	4-4.6		2	3	Yes	110				
Mayxay et al.	2004	The Lao PDR	Artesunate	Mefloquine	4		1	3	Yes	110				
Mayxay et al.	2006	The Lao PDR	Artesunate	Mefloquine	4		1	3	Yes	110				
Mayxay et al.	2006	The Lao PDR	Dihydroartemisinin	Piperaquine	2.1		1	3	Yes	110				
Menan et al.	2011	Cameroon, Ivory Coast and Seneg	Artemether	Lumefantrine	Not specified		2	3	Yes	197	43	10		
Menan et al.	2011	Cameroon, Ivory Coast and Seneg	Dihydroartemisinin	Piperaquine+Trimethoprim	3.2		2	2	Yes	206	41	3.9		
Ménard et al.	2007	Madagascar	Artesunate	Amodiaquine	4		1	3	Yes	83	67	11	2	
Ménard et al.	2008	Madagascar	Artesunate	Amodiaquine	4		1	3	Yes	346	62	12	2.9	PPR derived from figure
Mens et al.	2008	Kenya	Artemether	Lumefantrine	3.5-8		2	3	Partial	73		0	0	
Mens et al.	2008	Kenya	Dihydroartemisinin	Piperaquine	2.3-2.5		1	3	Yes	73		1.5	0	
Meremikwu et al.	2006	Nigeria	Artesunate	Amodiaquine	4		1	3	Yes	59				
Meremikwu et al.	2006	Nigeria	Artemether	Lumefantrine	3.5-8		2	3	Yes	60				
Michael et al.	2010	Nigeria	Artesunate	Amodiaquine	4		1	3	Yes	90	16	1	0	PPR derived from figure
Michael et al.	2010	Nigeria	Artemether	Lumefantrine	3.5-8		2	3	Yes	93	15	0	0	PPR derived from figure
Mockenhaupt et al.	2005	Ghana	Artesunate	Sulfadoxine+Pyrimethamine	4		1	3	Yes	145		20	2	PPR derived from figure
Mohamed et al.	2006	Sudan	Artemether	Lumefantrine	3		2	3	Yes	72		0	0	
Mohamed et al.	2006	Sudan	Artesunate	Sulfadoxine+Pyrimethamine	4		1	3	Yes	71		0	0	
Mukhtar et al.	2007	Sudan	Artemether	Lumefantrine	Not specified				Partial	80				
Mukhtar et al.	2007	Sudan	Artesunate	Sulfadoxine+Pyrimethamine	Not specified				Yes	80				
Mulenga et al.	2006	Zambia	Artemether	Lumefantrine		160	2	3	Partial	485			0.7	
Mutabingwa et al.	2005	Tanzania	Artesunate	Amodiaquine	4		1	3	No	515				
Mutabingwa et al.	2005	Tanzania	Artemether	Lumefantrine	4-4.6		2	3	No	519				
Na-Bangchang et al.	2010	Thailand	Artesunate	Mefloquine	4		1	3	Yes	150		0	0	
Nahum et al.	2007	Benin	Artesunate	Sulfadoxine+Pyrimethamine	4		1	3	Yes	81			5.1	
Nambei et al.	2005	Central Republic of Africa	Dihydroartemisinin	Piperaquine+Trimethoprim		96	3	1	Partial	54				
Nambozi et al.	2011	Zambia	Artemether	Lumefantrine	3.5-8		2	3	Yes	101				

Additional File 5 : Parasite Prevalence Rates

Authors	Year	Country	Artemisinin Derivatives	Partner Drug	Artemisinin Derivatives Dose (mg/kg/day)	Artemisinin Derivatives Dose (mg/day)	Doses Per Day	Days of Artemisinin	Supervised	Sample Size	D1 Parasitemic (%)	D2 Parasitemic (%)	D3 Parasitemic (%)	Comments
Nambozi et al.	2011	Zambia	Dihydroartemisinin	Piperaquine	2.25		1	3	Yes	203				
Ndayiragije et al.	2004	Burundi	Artesunate	Amodiaquine	4		1	3	Yes	153				
Ndayiragije et al.	2004	Burundi	Artemether	Lumefantrine	3.2-4		2	3	Yes	142				
Ndiaye et al.	2009	Cameroon, Madagascar, Mali anc	Artesunate	Amodiaquine	Not specified		2	3	Yes	315	61.8	7.7	0.6	
Ndiaye et al.	2009	Cameroon, Madagascar, Mali anc	Artesunate	Amodiaquine	Not specified		1	3	Yes	313	64.7	8.6	0	
Ndiaye et al.	2008	Senegal and Cameroon	Artesunate	Amodiaquine		50-200	2	3	Yes	155				
Ndiaye et al.	2008	Senegal and Cameroon	Artesunate	Amodiaquine		50-200	1	3	Yes	161				
Ndiaye et al.	2009	Cameroon, Madagascar, Mali anc	Artemether	Lumefantrine	Not specified		2	3	Yes	312	66.5	8.1	1.3	
Ngasala et al.	2011b	Tanzania	Artemether	Lumefantrine	3.3-8		2	3	No	179				Unsupervised
Ngasala et al.	2011a	Tanzania	Artemether	Lumefantrine	3.3-8		2	3	Partial	244				Unsupervised
Ngasala et al.	2011b	Tanzania	Artemether	Lumefantrine	3.3-8		2	3	Yes	180	71.3	6.7	1.1	
Nguyen et al.	2003	Vietnam	Artesunate	Chloroquine	2-4		1	3	Yes	28				
Nguyen et al.	2003	Vietnam	Artesunate	Chloroquine	2-4		1	3	Yes	33				
Nguyen et al.	2003	Vietnam	Artesunate	Sulfadoxine+Pyrimethamine	2-4		1	3	Yes	29				
Nguyen et al.	2003	Vietnam	Artesunate	Sulfadoxine+Pyrimethamine	2-4		1	3	Yes	33				
Noedl et al.	2006	Thailand	Artesunate	Azithromycin		200	2	3	Yes	27				
Noedl et al.	2006	Thailand	Artesunate	Azithromycin		200	1	3	Yes	27				
Noedl et al.	2010	Cambodia	Artesunate		4		1	7	Yes	60		47.9	21.9	
Obonyo et al.	2003	Kenya	Artesunate	Sulfadoxine+Pyrimethamine	4		1	1	Yes	200	64	20	4	PPR derived from figure
Obonyo et al.	2003	Kenya	Artesunate	Sulfadoxine+Pyrimethamine	4		1	3	Yes	200	64	10	2	PPR derived from figure
Oduro et al.	2008	Ghana	Artesunate	Amodiaquine	4		1	3	No	154			0	Unsupervised; PPR derived from
Oduro et al.	2008	Ghana	Artesunate	Amodiaquine	4		1	3	Yes	154			0	Supervised; PPR derived from figure
Oduro et al.	2004	Ghana	Beta-artemether			80-240	1-2	4	Partial	117			7	
Oduro et al.	2004	Ghana	Beta-artemether			80-160	1-2	5	Partial	106				
Osorio et al.	2007	Colombia	Artesunate	Amodiaquine	4		1	3	Yes	42				
Owusu-Agyei et al.	2008	Ghana	Artesunate	Amodiaquine	Not specified				Yes	178				
Owusu-Agyei et al.	2008	Ghana	Artesunate	Chlorproguanil+Dapsone	Not specified				Yes	178				
Owusu-Agyei et al.	2008	Ghana	Artemether	Lumefantrine	Not specified				Yes	177				
Oyakhirome et al.	2007	Gabon	Artesunate	Amodiaquine	4		1	3	No	32				Unsupervised
Oyakhirome et al.	2007	Gabon	Artesunate	Amodiaquine	4		1	3	Yes	29				Supervised
Penali et al.	2008	Ivory Coast	Artesunate	Sulfamethoxypyrazine+Pyrimethamine	5-10		1	3	Yes	111		0	0	
Penali et al.	2008	Ivory Coast	Artesunate	Sulfamethoxypyrazine+Pyrimethamine	5-20		1-2	2	Yes	110		0	0	
Piola et al.	2005	Uganda	Artemether	Lumefantrine	4-4.6		2	3	Partial	615				Unsupervised
Piola et al.	2005	Uganda	Artemether	Lumefantrine	4-4.6		2	3	Yes	313				Supervised
Premji et al.	2009	Burkina Faso, Ghana, Kenya, Nige	Artesunate	Chlorproguanil+Dapsone	4		1	3	Yes	914				
Premji et al.	2009	Burkina Faso, Ghana, Kenya, Nige	Artemether	Lumefantrine	4.6-8		2	3	Yes	458				
Priotto et al.	2003	Uganda	Artesunate	Sulfadoxine+Pyrimethamine	4		1	1	Yes	126	95	50	36.8	
Priotto et al.	2003	Uganda	Artesunate	Sulfadoxine+Pyrimethamine	4		1	3	Yes	126	86	28	3.5	
Pukrittayakamee et al.	2004	Thailand	Artesunate	Primaquine	1.65-3.3		1	7	Yes	27				
Pukrittayakamee et al.	2004	Thailand	Artesunate		1.65-3.3		1	7	Yes	23				
Rahman et al.	2008	Bangladesh	Artemether	Lumefantrine	4.6-8		2	3	No	160				Unsupervised
Rahman et al.	2008	Bangladesh	Artemether	Lumefantrine	4.6-8		2	3	Yes	160				Supervised
Ramharter et al.	2005	Gabon	Artesunate	Clindamycin	4		2	3	Yes	50				
Rasheed et al.	2011	Liberia	Artemether	Lumefantrine	4.6-8		2	3	Yes	100				
Ratcliff et al.	2007	Indonesia	Artemether	Lumefantrine	4-4.6		2	3	Partial	375	43	3		
Ratcliff et al.	2007	Indonesia	Dihydroartemisinin	Piperaquine	2.25		1	3	Yes	379	27	3		
Rojanawatsirivej et al.	2003	Thailand	Artemether	Lumefantrine+Primaquine	4.6		2	3	Not stated	33				
Rojanawatsirivej et al.	2003	Thailand	Artesunate	Mefloquine+Primaquine	4		1	3	Not stated	46			2.17	
Rojanawatsirivej et al.	2003	Thailand	Artesunate	Mefloquine+Primaquine	4		1	3	Not stated	153				
Rulisa et al.	2007	Rwanda	Artesunate	Sulfadoxine+Pyrimethamine	4		1	3	Yes	103				
Rulisa et al.	2007	Rwanda	Artesunate	Sulfamethoxypyrazine+Pyrimethamine	4		1	3	Yes	109				
Rwagacondo et al.	2004	Rwanda	Artesunate	Amodiaquine	4		1	3	Yes	158				
Rwagacondo et al.	2003	Rwanda	Artesunate	Sulfadoxine+Pyrimethamine	4		1	3	Yes	144				
Sagara et al.	2006	Mali	Artemether	Lumefantrine	4.6-8		1-2	3	Yes	303		2.6	0	
Sagara et al.	2008	Mali	Artemether	Lumefantrine	4.6-8		2	3	Yes	235	67.9	2.1	0	
Sagara et al.	2009	Cameroon, Mali, Rwanda and Su	Artemether	Lumefantrine	4.6-8		2	3	Yes	450	48.2	9.2	0.8	
Sagara et al.	2008	Mali	Artesunate	Mefloquine	4		1	3	Yes	235	65.2	2.6	0	
Sagara et al.	2006	Mali	Artesunate	Sulfamethoxypyrazine+Pyrimethamine	4		1	3	Yes	303		0.7	0.7	
Sagara et al.	2009	Cameroon, Mali, Rwanda and Su	Artesunate	Sulphamethoxypyrazine+Pyrimethamine	3.8-10		1-2	2	Yes	458	38.8	9	1	

Additional File 5 : Parasite Prevalence Rates

Authors	Year	Country	Artemisinin Derivatives	Partner Drug	Artemisinin Derivatives Dose (mg/kg/day)	Artemisinin Derivatives Dose (mg/day)	Doses Per Day	Days of Artemisinin	Supervised	Sample Size	D1 Parasitemic (%)	D2 Parasitemic (%)	D3 Parasitemic (%)	Comments
Sagara et al.	2009	Cameroon, Mali, Rwanda and Su	Artesunate	Sulphamethoxypyrazine+Pyrimethamine	3.8-5		1	3	Yes	476	52.5	9.5	0.5	
Schwarz et al.	2005	Gabon	Artesunate		2-4		1	5	Partial	50				
Silachamroon et al.	2005	Thailand	Artesunate	Mefloquine	4		1	3	Yes	60			0	
Silachamroon et al.	2005	Thailand	Artesunate	Mefloquine	4		1	3	Yes	60			0	
Sirima et al.	2009	Burkina Faso	Artesunate	Amodiaquine	4		1	3	Yes	375		2		Loose
Sirima et al.	2009	Burkina Faso	Artesunate	Amodiaquine	4		1	3	Yes	375		2.3		Fixed Dose
Sirima et al.	2003	Burkina Faso	Artesunate	Chloroquine	4		1	3	Yes	150	78	20	4	PPR derived from figure
Sirivichayakul et al.	2007	Thailand	Artesunate	Mefloquine	10		1	3	Yes	35			0	Rectal
Sirivichayakul et al.	2007	Thailand	Artesunate	Mefloquine	20		1	3	Yes	35			0	Rectal
Smithuis et al.	2010	Myanmar	Artesunate	Amodiaquine	4		1	3	Partial	155				
Smithuis et al.	2010	Myanmar	Artemether	Lumefantrine	3.3		2	3	Partial	162				
Smithuis et al.	2006	Myanmar	Artesunate	Mefloquine	4		1	3	No	162				Unsupervised
Smithuis et al.	2004a	Myanmar	Artesunate	Mefloquine	4		1	3	No	196				
Smithuis et al.	2010	Myanmar	Artesunate	Mefloquine	4		1	3	Partial	161				Loose
Smithuis et al.	2010	Myanmar	Artesunate	Mefloquine	4		1	3	Partial	169				Fixed Dose
Smithuis et al.	2006	Myanmar	Artesunate	Mefloquine	4		1	3	Yes	162	55	6		Supervised
Smithuis et al.	2004a	Myanmar	Artesunate	Mefloquine	4		1	3	Yes	198				Supervised
Smithuis et al.	2004a	Myanmar	Artesunate	Mefloquine	4		1	1	Yes	203				MQ15 single dose
Smithuis et al.	2004a	Myanmar	Artesunate	Mefloquine	4		1	1	Yes	206				MQ25 single dose
Smithuis et al.	2004b	Myanmar	Artesunate	Mefloquine	4		1	1	Yes	78			5	
Smithuis et al.	2006	Myanmar	Dihydroartemisinin	Piperaquine	2.1		1	3	No	171				Unsupervised
Smithuis et al.	2006	Myanmar	Dihydroartemisinin	Piperaquine	2.1		1	3	Yes	156	47	3		Supervised
Smithuis et al.	2010	Myanmar	Dihydroartemisinin	Piperaquine	2.5		1	3	Partial	161				
Song et al.	2011	Cambodia	Artemether	Lumefantrine		160	2	3	Yes	55				
Song et al.	2011	Cambodia	Dihydroartemisinin	Piperaquine		160	2	2	Yes	55				
Song et al.	2011	Cambodia	Artemisinin	Piperaquine		125	1	2	Yes	110				
Sowunmi et al.	2005	Nigeria	Artesunate	Amodiaquine	4		1	3	Yes	104	72.8	0	0	
Sowunmi et al.	2007a	Nigeria	Artesunate	Amodiaquine	4		1	3	Yes	120				
Sowunmi et al.	2011	Nigeria	Artesunate	Amodiaquine	5.5		1	3	Yes	60	10	0	0	Coformulated
Sowunmi et al.	2011	Nigeria	Artesunate	Amodiaquine	4.4-10		1	3	Yes	60	5	0	0	Copackaged
Sowunmi et al.	2007b	Nigeria	Artemether	Lumefantrine	4.6-8		2	3	Partial	90	61.1	6.7		
Sowunmi et al.	2009	Nigeria	Artesunate	Mefloquine	4		1	3	Yes	171	36.8	6.4		
Sowunmi et al.	2007a	Nigeria	Artesunate		4		1	7	Yes	120				
Staedke et al.	2004	Uganda	Artesunate	Amodiaquine	4		1	3	Yes	130	55	3	0	PPR derived from figure
Stohrer et al.	2004	The Lao PDR	Artemether	Lumefantrine	3.4-4		2	3	Yes	53	46.8	4.3	0	
Stohrer et al.	2004	The Lao PDR	Artesunate	Mefloquine	4		1	3	Yes	55	28.3	1.9	0	
Suputtamongkol et al.	2003	Thailand	Artesunate	Mefloquine	4		1	3	Not stated	320				
Sutherland et al.	2003	The Gambia	Artesunate	Chloroquine	4		1	3	Yes	400				
Sutherland et al.	2005	The Gambia	Artemether	Lumefantrine	4.8-12		2	3	Yes	406				
Swarthout et al.	2006	The Democratic Republic of Cong	Artesunate	Amodiaquine	Not specified			3	Yes	90			0	
Swarthout et al.	2006	The Democratic Republic of Cong	Artesunate	Sulfadoxine+Pyrimethamine	Not specified			3	Yes	90			0	
Sykes et al.	2009	Tanzania	Artesunate	Azithromycin	4		1	3	Yes	129				
Sykes et al.	2009	Tanzania	Artemether	Lumefantrine		40-80	2	3	Partial	132				
Tall et al.	2007	The Comoros Union	Artesunate	Amodiaquine	Not specified				Yes	54		22.5		
Tall et al.	2007	The Comoros Union	Artesunate	Sulfadoxine+Pyrimethamine	Not specified				Yes	53		13.2		
Tangpukdee et al.	2005	Thailand	Artesunate	Mefloquine	4		1	3	Yes	60				
Tangpukdee et al.	2008	Thailand	Artesunate	Mefloquine	4		1	3	Yes	65		3.07		
Tangpukdee et al.	2005	Thailand	Dihydroartemisinin	Piperaquine	2		1	3	Yes	120				
Tangpukdee et al.	2008	Thailand	Artemisinin	Piperaquine+Primaquine	3.2		1	3	Yes	65		4.6		
Thanh et al.	2009	Vietnam	Artesunate	Amodiaquine	4.4		1	3	Yes	56				
Thanh et al.	2009	Vietnam	Dihydroartemisinin	Piperaquine	2.3		1	3	Yes	60				
Thapa et al.	2007	Nepal	Artemether	Lumefantrine	4		2	3	Yes	66				
Thriemer et al.	2010	Bangladesh	Artesunate	Azithromycin	4		1	3	Yes	152	63.21	0	0	
Thriemer et al.	2010	Bangladesh	Artemether	Lumefantrine	4		2	3	Yes	76	63.21	0	0	
Thwing et al.	2009	Kenya	Artesunate	Amodiaquine	4		1	3	Yes	110	70	15.4	1.8	
Tietche et al.	2010	Cameroon	Artesunate	Mefloquine	2.5-5		1	3	Partial	213				
Tiono et al.	2009	Burkina Faso, Ghana, Mali, Nigeri	Artesunate	Chlorproguanil+Dapsone	4		1	3	Yes	600	48			
Tjitra et al.	2001	Indonesia	Artesunate	Sufadoxine+Pyrimethamine	4		1	3	Yes	53	39.2	0	0	
Toure et al.	2011	Ivory coast	Artemether	Lumefantrine	4.6-8		2	3	Not stated	79		1	0	

Additional File 5 : Parasite Prevalence Rates

Authors	Year	Country	Artemisinin Derivatives	Partner Drug	Artemisinin Derivatives Dose (mg/kg/day)	Artemisinin Derivatives Dose (mg/day)	Doses Per Day	Days of Artemisinin	Supervised	Sample Size	D1 Parasitemic (%)	D2 Parasitemic (%)	D3 Parasitemic (%)	Comments
Toure et al.	2009	Ivory Coast	Artemether	Lumefantrine	4.6-8		2	3	Partial	61	31.1	0	0	
Toure et al.	2011	Ivory coast	Artesunate	Mefloquine		50	1	3	Not stated	77		1	0	Paediatric Formulation
Toure et al.	2009	Ivory Coast	Artemisinin	Naphthoquine	20.9-28.6		2	1	Partial	62	29	0	0	
Trung et al.	2009	Vietnam	Dihydroartemisinin	Piperaquine	Not specified		2	2	Yes	51				
Trung et al.	2009	Vietnam	Artemisinin	Piperaquine		62.5-125	1	2	Yes	52				
Tshefu et al	2010	The Democratic Republic of Cong	Artemether	Lumefantrine	1.8-4		2	3	Yes	423	48	4.7		
Tshefu et al	2010	The Democratic Republic of Cong	Artesunate	Pyronaridine	2.4-4.6		1	3	Yes	849	34	4.5		
Tun et al.	2009	Myanmar	Artemisinin	Naphthoquine		1000	1	1	Yes	53				
Ursing et al.	2011	Guinea-Bissau	Artemether	Lumefantrine		40-160	2	3	Yes	190				
Valecha et al.	2009	India	Artemether	Lumefantrine	4-4.6		2	3	Yes	71		9.9		
Valecha et al.	2009	India	Artemether	Lumefantrine	4-4.6		2	3	Yes	53		20.8		
Valecha et al.	2010b	Thailand, The Lao PDR and India	Artesunate	Mefloquine	4		1	3	Yes	381			2.4	
Valecha et al.	2010b	Thailand, The Lao PDR and India	Dihydroartemisinin	Piperaquine	2.25		1	3	Yes	767			2.4	
Valecha et al.	2010a	Thailand, India, and Tanzania	Arterolane			100	1	7	Yes	76				
Valecha et al.	2010a	Thailand, India, and Tanzania	Arterolane			200	1	7	Yes	76				
Valecha et al.	2010a	Thailand, India, and Tanzania	Arterolane			50	1	7	Yes	78				
van den Broek et al.	2006	The Democratic Republic of Cong	Artesunate	Amodiaquine	4		1	3	Yes	101			0	
van den Broek et al.	2005a	Sudan	Artesunate	Amodiaquine	4		1	3	Yes	134		17		
van den Broek et al.	2006	The Democratic Republic of Cong	Artemether	Lumefantrine	Not specified		2	3	Yes	106			0	
van den Broek et al.	2005b	Bangladesh	Artemether	Lumefantrine	Not specified		2	3	Yes	121				
van den Broek et al.	2005b	Bangladesh	Artesunate	Mefloquine	4		1	3	Yes	121				
van den Broek et al.	2006	The Democratic Republic of Cong	Artesunate	Sufadoxine+Pyrimethamine	4		1	3	Yes	91			0	
van den Broek et al.	2005a	Sudan	Artesunate	Sufadoxine+Pyrimethamine	4		1	3	Yes	135		22		
van Vugt et al.	2002	Thailand	Artesunate	Atovaquone+Proguanil	4		1	3	Yes	533			0.4	
van Vugt et al.	2000	Thailand	Artemether	Lumefantrine	3.2		2	3	Yes	150	76	8	1	PPR derived from figure
van Vugt et al.	2000	Thailand	Artesunate	Mefloquine	4		1	3	Yes	50	74	10	0	PPR derived from figure
van Vugt et al.	2002	Thailand	Artesunate	Mefloquine	4		1	3	Yes	533			0.4	
Vasquez et al.	2009	Colombia	Artesunate	Mefloquine		50-200	1	3	Not stated	25	50	4	0	
Vasquez et al.	2009	Colombia	Artesunate	Mefloquine+Primaquine		50-200	1	3	Not stated	25	50	0	0	
von Seidlein et al.	2000	The Gambia	Artesunate	Sulfadoxine+Pyrimethamine	4		1	1	Yes	200	47	8	0.5	
von Seidlein et al.	2000	The Gambia	Artesunate	Sulfadoxine+Pyrimethamine	4		1	3	Yes	200	47	1	0	
von Seidlein et al.	2001	The Gambia	Artesunate	Sulfadoxine+Pyrimethamine	4		1	3	Yes	74				
von Seidlein et al.	2001	The Gambia	Artesunate	Sulfadoxine+Pyrimethamine	4		1	1	Yes	113				
Wang et al.	2001	Equatorial Guinea	Dihydroartemisinin	Mefloquine		120	1	1	Not stated	54				
Warsame et al.	2009	Somalia	Artesunate	Amodiaquine	4		1	3	Yes	198				
Warsame et al.	2009	Somalia	Artesunate	Sufadoxine+Pyrimethamine	4		1	3	Yes	293				
Wattanakoon et al.	2003	Thailand	Artesunate	Mefloquine		100-150	1	2	Not stated	516		5		
Weerasinghe et al.	2002	Sri Lanka	Artesunate	Sulphadoxine+Pyrimethamine+Primaquin	4		1	3	Yes	30	20	3		
Whegang et al.	2010	Cameroon	Artesunate	Amodiaquine	4		1	3	Yes	278			1.3	
Whegang et al.	2010	Cameroon	Artesunate	Chlorproguanil+Dapsone	4		1	3	Yes	83			2.4	
Whegang et al.	2010	Cameroon	Artemether	Lumefantrine	Not specified		2	3	Yes	61			0	
Whegang et al.	2010	Cameroon	Artesunate	Mefloquine	4		1	3	Yes	69			4.3	
Whegang et al.	2010	Cameroon	Dihydroartemisinin	Piperaquine	2.1		1	3	Yes	91			0	
Whegang et al.	2010	Cameroon	Artesunate	Sulfadoxine+Pyrimethamine	4		1	3	Yes	146			3.2	
Wong et al.	2003	Malaysia	Artemisinin	Beta cyclodextrin		300	2	5	Yes	50				
Wong et al.	2003	Malaysia	Artemisinin			500	2	5	Yes	50				
Wootton et al.	2008	Malawi and The Gambia	Artesunate	Chlorproguanil+Dapsone	1		1	3	Yes	47				
Wootton et al.	2008	Malawi and The Gambia	Artesunate	Chlorproguanil+Dapsone	2		1	3	Yes	45				
Wootton et al.	2008	Malawi and The Gambia	Artesunate	Chlorproguanil+Dapsone	4		1	3	Yes	40				
Yavo et al.	2011	Cameroon, Ivory Coast and Seneg	Artemether	Lumefantrine	4.6-8		2	3	Yes	187	43	5.9		
Yavo et al.	2011	Cameroon, Ivory Coast and Seneg	Dihydroartemisinin	Piperaquine	2.4-4		1	3	Yes	197	42.9	4.7		
Yeka et al.	2005	Uganda	Artesunate	Amodiaquine	4		1	3	Yes	731		5.5		
Yeka et al.	2008	Uganda	Artemether	Lumefantrine	4.6-8		2	3	Yes	199		2.5	0	
Yeka et al.	2008	Uganda	Dihydroartemisinin	Piperaquine	2.1		1	3	Yes	215		3.3	0	
Zongo et al.	2007a	Burkina Faso	Artemether	Lumefantrine	4.6-8		2	3	Yes	261		4.6	1.6	
Zongo et al.	2007b	Burkina Faso	Artemether	Lumefantrine	4.6-8		2	3	Yes	188		3	0	
Zongo et al.	2007b	Burkina Faso	Dihydroartemisinin	Piperaquine	6.4		1	3	Yes	187		3	0	
Zoungrana et al.	2008	Burkina Faso	Artesunate	Amodiaquine	4		1	3	Yes	61		21.3	4.9	
Zoungrana et al.	2008	Burkina Faso	Artesunate	Methylene blue	4		1	3	Yes	61		11.5	1.6	
