# Supplementary figures and images for: Early parasitological response following artemisinin-containing regimens: a critical review of the literature
Source: Malar J. 2013 Apr 19;12:125. doi: 10.1186/1475-2875-12-125 (PMC3649884; doi:10.1186/1475-2875-12-125)

## Slide 1
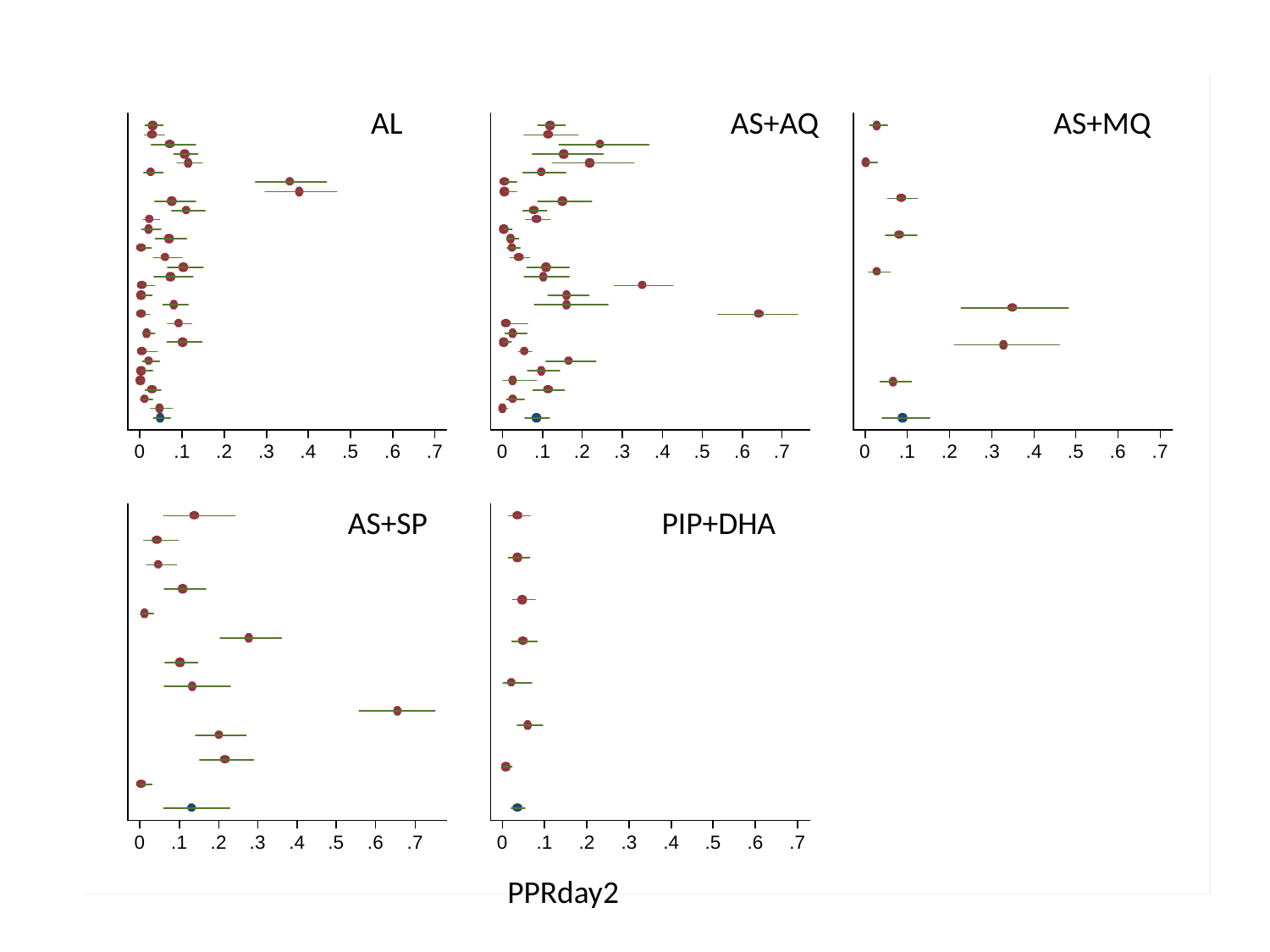

AL
 AS+AQ
 AS+MQ
 AS+SP
 PIP+DHA
PPRday2

Supplement: Additional file 6 — Forest plots of Day 2 parasite positivity rates in Africa. Estimates and 95% CI are shown by treatment, sorted by year in descending order (most recent first). Heterogeneity between studies I2 : AL = 92.3%; AS+AQ = 92.9%; AS+MQ = 91.7%; AS+SP = 95.6%; PIP+DHA = 60.9%. [file 1475-2875-12-125-S6.pptx]

## Slide 1
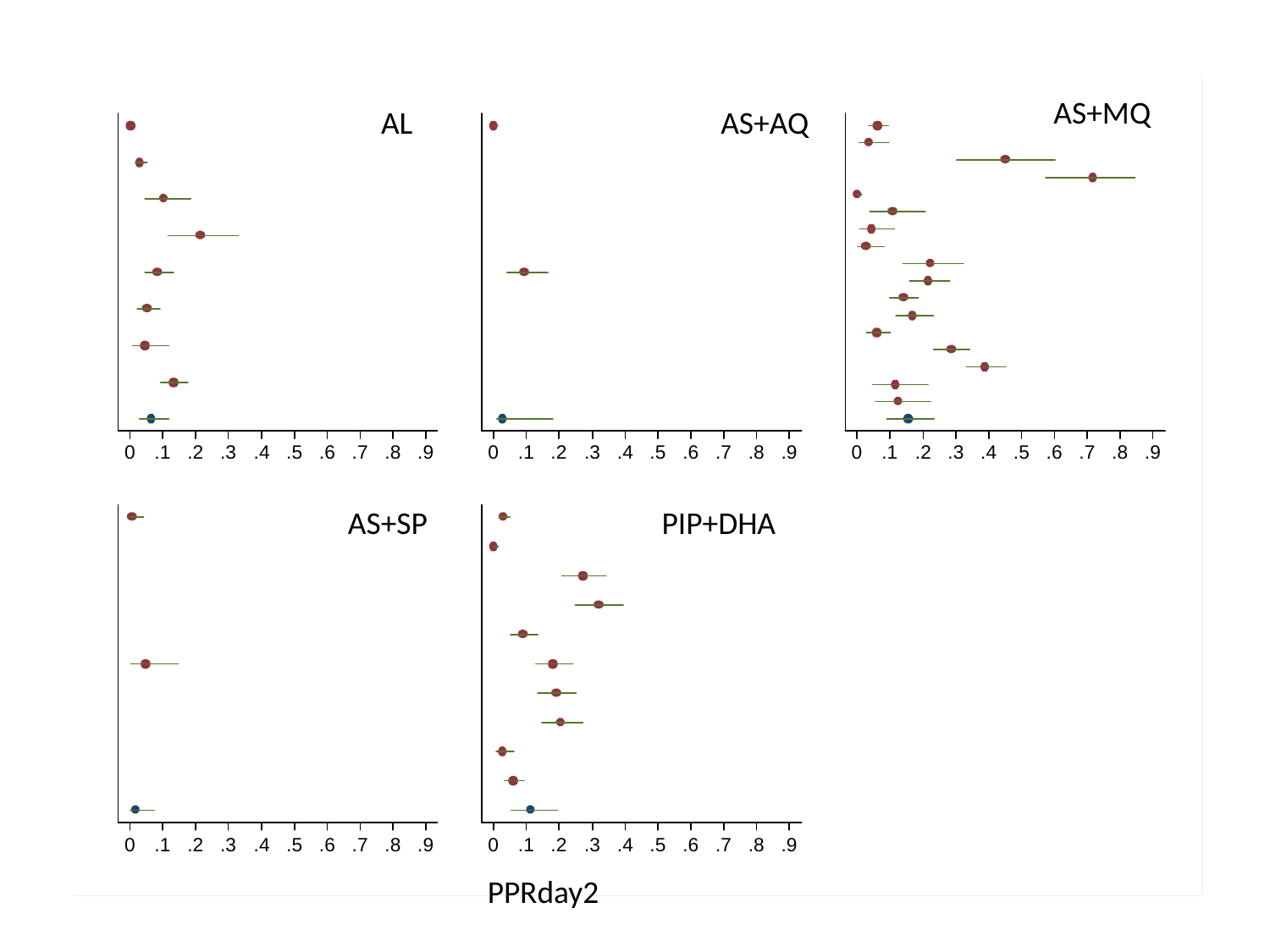

AS+MQ
 AL
 AS+AQ
 AS+SP
 PIP+DHA
PPRday2

Supplement: Additional file 7 — Forest plots of Day 2 parasite positivity rates in Asia. Estimates and 95% CI are shown by treatment, sorted by year in descending order (most recent first). Heterogeneity between studies I2 : AL = 90.3%; AS+AQ = 93.8%; AS+MQ = 95.3%; AS+SP = 42.1%; PIP+DHA = 96.0%. [file 1475-2875-12-125-S7.pptx]
