# Supplementary material for: Early parasitological response following artemisinin-containing regimens: a critical review of the literature
Source: Malar J. 2013 Apr 19;12:125. doi: 10.1186/1475-2875-12-125 (PMC3649884; doi:10.1186/1475-2875-12-125)
Supplement: Additional file 8 — Studies with Day 3 parasitaemia positivity rates ≥10%. [file 1475-2875-12-125-S8.pdf]

Authors	Study site	Drug evaluated	Dose per day - ART X days	Sample size	D3 parasitaemic (%)	Comment
Dondorp et al. 2009	Pailin, Cambodia	AS+MQ	4 mg/kg/day X 3	20	55	Area of known artemisinin tolerance
Dondorp et al. 2009	Pailin, Cambodia	AS	2 mg/kg/day X 7	20	55	Area of known artemisinin tolerance
Priotto et al. 2003	Mbarara, Uganda	AS+SP	4 mg/kg/day single dose	126	36.8	Single dose artesunate
Adjuik et al. 2002	Mlomp, Senegal	AS+AQ	4 mg/kg/day X 3	160	10	Rate derived from publication figure
Noedl et al. 2010	Tasanh, Cambodia	AS	4 mg/kg/day X 7	60	21.9	Area of known artemisinin tolerance
Puji et al. 2009	West Sumba District, Indonesia	AS+AQ	4 mg/kg/day X 3	103	12.6	Parasitaemia defined by microscopy and PCR
Bethell et al. 2011	Tasanh, Cambodia	AS	2 mg/kg/day X 7	75	49	Area of known artemisinin tolerance
Bethell et al. 2011	Tasanh, Cambodia	AS	4 mg/kg/day X 7	40	46	Area of known artemisinin tolerance
Bethell et al. 2011	Tasanh, Cambodia	AS	6 mg/kg/day X 7	28	48	Area of known artemisinin tolerance
